# Bidirectional Chemo-Mechanical Interface Stabilization in Perovskite Solar Cells

**DOI:** 10.1021/jacs.6c06932

**Published:** 2026-07-16

**Authors:** Qian Cheng, Xiaofen Li, Mingwei Hao, Kuan Wang, Jiahong Tang, Pengfei Guo, Lifei He, Wenjian Yu, Changyu Yang, Du Chen, Peijun Guo, Yuanyuan Zhou

**Affiliations:** † Department of Chemical and Biological Engineering, 58207 The Hong Kong University of Science and Technology, Clear Water Bay, Hong Kong SAR 00000, China; ‡ Department of Chemical and Environmental Engineering, 5755 Yale University, New Haven, Connecticut 06520 United States; § Energy Sciences Institute, Yale University, West Haven, Connecticut 06516 United States; ∥ Energy Institute, The Hong Kong University of Science and Technology, Clear Water Bay, Hong Kong SAR 00000, China; ⊥ Otto Poon Center for Climate Resilience and Sustainability, The Hong Kong University of Science and Technology, Clear Water Bay, Hong Kong SAR 00000, China

## Abstract

The interface structures between dissimilar layers in perovskite solar cells (PSCs) are prone to the concurrent occurrence of lateral (in-plane) chemical aggregation and vertical (out-of-plane) mechanical delamination. This issue severely affects long-term optoelectronic processes in PSCs, and it has not been addressed holistically. Herein, we introduce an ultrathin interfacial layer of 1,3,6,8-pyrenetetrasulfonic tetrasodium salt (PTS) to stabilize the perovskite/C_60_ interface at the molecular level. The sulfonate groups in PTS molecules anchor to the perovskite interface, while the parallelly aligned pyrene cores establish robust π–π interactions with C_60_ molecules. The reconstructed interface enhances the interfacial adhesion and restricts the mobility of C_60_ molecules, enabling a bidirectional chemo-mechanical interface stabilization (BCIS) mechanism at the perovskite/C_60_ interface. The resultant PSCs deliver power conversion efficiencies (PCEs) of up to 26.53%, showing 96% PCE retention after 1,000 h maximum-power-point tracking (ISOS-L-1l), and 91% PCE retention after 300 thermal cycles (−40 to 85 °C, IEC61215 MQT11). The scalability of PTS treatment is demonstrated by the 818 cm^2^ (aperture area) perovskite solar modules (PSMs) with PCEs over 20% using industrial-compatible manufacturing processes under 55% relative humidity (RH). This work underscores bidirectional interface engineering as a critical strategy for advancing commercially viable perovskite photovoltaics.

## Graphical abstract

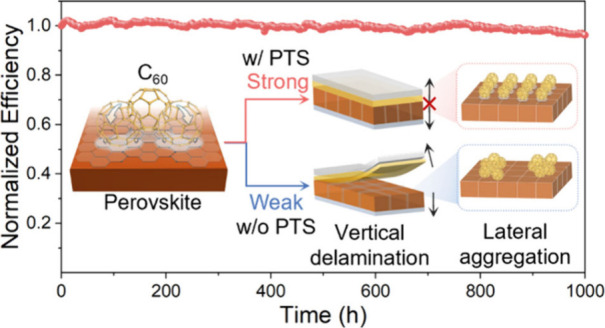

## Introduction

Perovskite solar cells (PSCs) have emerged as one of the most promising next-generation photovoltaic technologies due to their exceptional optoelectronic properties, solution-processability, and rapid improvements in power conversion efficiency (PCE). In particular, inverted PSC architectures (*p-i-n* configuration) have garnered significant attention due to their superior compatibility with tandem applications and scalable processing.^1–3^ The swift increase in certified PCEs for both inverted perovskite single cells and modules underscores their potential for industrial deployment.^4^ Nevertheless, operational stability and mechanical reliability continue to hinder the commercialization of PSCs.^5,6^ The interfacial degradation originates from the weak interactions between the perovskite layer and the charge transport layers.^7–10^ While substantial research has been dedicated to stabilizing the perovskite layer itself and its buried bottom interface,^11–16^ comparatively fewer efforts have focused on the upper perovskite/C_60_ interface, which is especially vulnerable due to its inherently weak adhesion and lack of reliable molecular bonding.^17–20^ This unreliable interface faces two major degradation pathways: (i) lateral C_60_ aggregation, in which aggregated C_60_ molecules induce microstructural and energetic disorder, increasing interfacial nonradiative recombination. (ii) vertical mechanical delamination, where localized separation reduces the effective area for charge extraction and initiates irreversible damage propagation. These phenomena could degrade device performance in a detrimental feedback loop under external stresses.

In this work, we utilize a bifunctional interlayer of 1,3,6,8-pyrenetetrasulfonic tetrasodium salt (PTS) to unlock a bidirectional chemo-mechanical stabilization of the perovskite/C_60_ interface. PTS features a conjugated pyrene core functionalized with four sulfonate groups in a symmetric configuration. Driven by the anchoring capability of the sulfonate groups, the pyrene units are aligned parallel to the perovskite grain surfaces, enabling strong π–π stacking interactions with C_60_ molecules. This configuration not only reinforces interfacial adhesion but also creates an interfacial microstructure that inhibits lateral migration and aggregation of C_60_ molecules. In addition to the bidirectional chemo-mechanical interface stabilization (BCIS) effect, PTS also enhances electronic coupling between the perovskite and C_60_ layers, thereby facilitating efficient charge extraction and suppressing interfacial recombination. Our BCIS mechanism could effectively mitigate both vertical delamination and lateral aggregation while minimizing the interfacial energy losses in inverted PSCs.

The resultant PSCs exhibited improved PCE and stability. Small-area PSCs achieve PCEs of up to 26.53%, and their stability tests show 96% efficiency retention after 1,000 h of operation upon maximum-power-point tracking under one-sun illumination (ISOS-L-1l protocol) and 91% after 300 thermal cycles between −40 and 85 °C (IEC61215:2021 MQT11 protocol). Importantly, the PTS strategy is scalable and compatible with industrial manufacturing. We applied the PTS treatment to perovskite solar modules (PSMs) fabricated using industrial-compatible slot-die coating processes. A PCE of 20.08% with an aperture area of 818 cm^2^ was achieved under a high relative humidity (RH) of 55%. We further validated the generality of the BCIS concept through experiments spanning different perovskite compositions, interfacial materials, and device structures. This study emphasizes the importance of holistic interface engineering, encompassing both electronic and stability considerations, to improve the performance of perovskite photovoltaics for real-world deployment.

## Results and Discussion

### Bidirectional, Chemo-Mechanical Failure of the Perovskite/C_60_ Interface

As previously mentioned, the perovskite/C_60_ interface exhibits lateral C_60_ aggregation and vertical delamination under external stress (Figure 1A). We first investigated the aggregation behavior of C_60_ under photothermal aging based on a series of characterizations. The samples were continuously heated at 85 °C under white LED illumination for 7 days in N_2_ atmosphere. In the aged control sample, the full coverage of the C_60_ layer is disrupted, indicating degraded interfacial integrity and electrical properties of the perovskite/C_60_ interface (Figure 1B and Figure S1). Similarly, the atomic force microscopy (AFM) images of the aged control sample (Figure S2) reveal distinct micrometer-scale protrusions on the surface. Moreover, comparative AFM analysis of the bare perovskite layers (Figure S3) confirms that these protrusions are more likely to originate from the C_60_ layer rather than the perovskite itself. In contrast, the C_60_ layer in the target sample remains continuous and uniform (Figure 1C), exhibiting exceptional stability under photothermal aging conditions.

**Figure 1:**
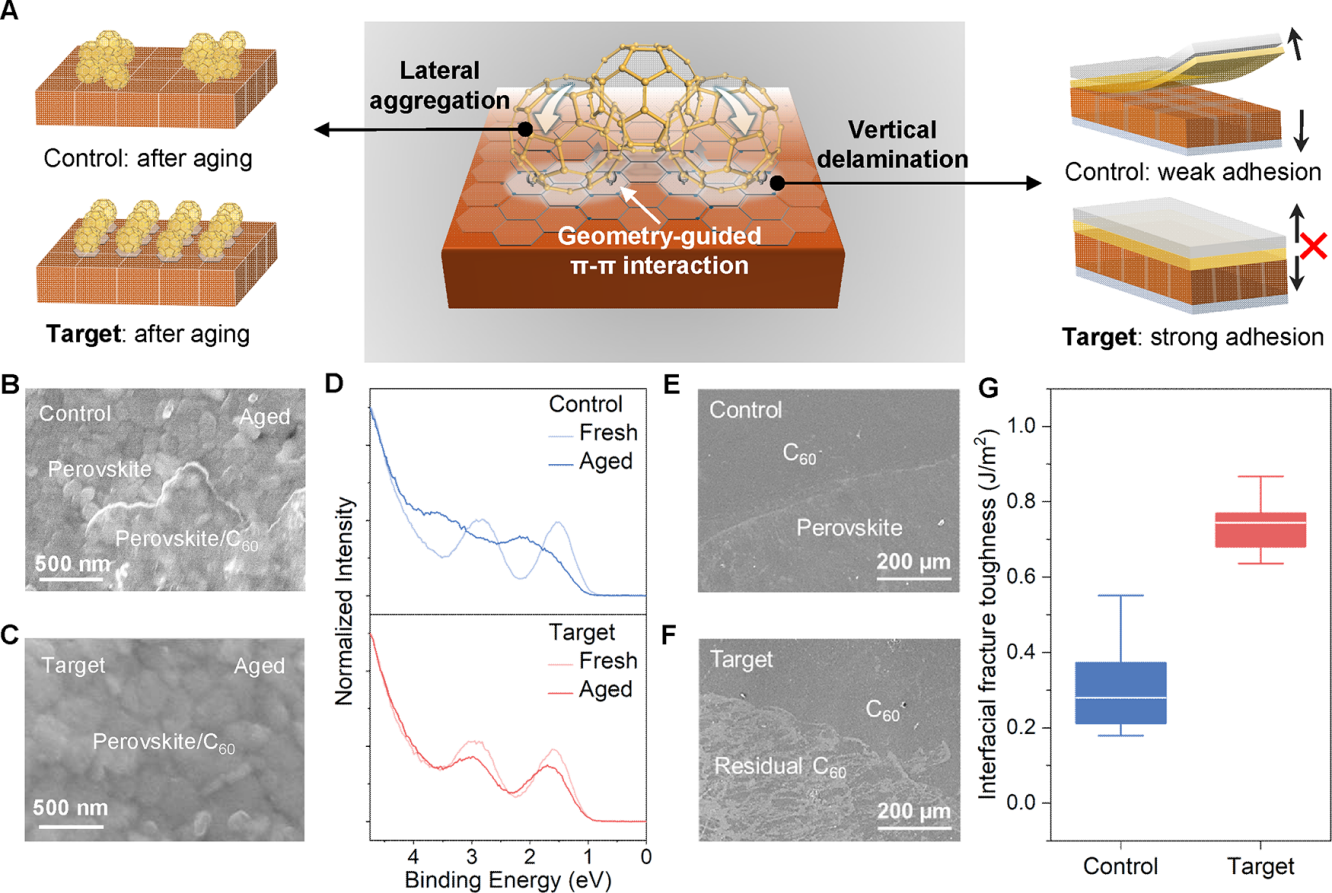
Bidirectional chemo-mechanical interfacial stabilization (BCIS) induced by PTS. (A) Schematic illustration showing the degradation of the perovskite/C_60_ interface and the effect of the PTS. The samples are continuously heated at 85 °C under white LED illumination for 7 days in N_2_ atmosphere to complete the aging process. (B, C) SEM images of the aged control and target perovskite/C_60_ films. (D) UPS spectra of control and target perovskite/C_60_ films before and after aging. (E, F) SEM images showing the surface of perovskite single crystals/C_60_ after mechanical delamination. (G) Interfacial fracture toughness of the perovskite/C_60_ interface with and without the PTS interlayer based on double cantilever beam (DCB) tests. Control: the film without the PTS interlayer. Target: the film with the PTS interlayer.

Further evidence comes from the analysis of ultraviolet photoelectron spectroscopy (UPS) spectra (Figure 1D). In fresh samples, C_60_ shows clear peaks corresponding to the highest occupied molecular orbitals (HOMO and HOMO–1), which feature significant energetic changes after photothermal aging. As shown in Figure S4, these peaks of the control sample shift by 0.4 and 0.8 eV toward higher binding energies, respectively. The pronounced peak broadening and attenuation indicate increased energy disorder and C_60_ aggregation.^21,22^ A similar conclusion could be drawn from the UV–vis absorption spectra (Figure S5), where the shifted and broadened absorption peaks are also observed in the control sample.^23–26^ In contrast, the UPS and UV–vis absorption peaks of the target sample show only minor shifts and broadening, indicating that PTS effectively suppresses C_60_ aggregation. To verify the generality of our strategy, we extend our investigation to PCBM, a commonly used C_60_ derivative as the ETL in PSCs. After photothermal aging, the control sample exhibits significant cracking (Figure S6) and more pronounced changes in its absorption peaks (Figure S7), likely due to PCBM’s higher tendency to aggregate compared to C_60_. With PTS treatment, PCBM aggregation is also effectively suppressed, thereby maintaining the film properties in aged samples.

Regarding the mechanical failure, the delamination typically occurs at the weaker interface when external force is applied. To minimize interference from other interfaces, we deposited C_60_ onto polished MAPbBr_3_ single crystals and peeled it off with adhesive tapes. Scanning electron microscopy (SEM) images show that the C_60_ layer on the control sample is cleanly peeled off with a clear boundary, while significant C_60_ residue remains on the PTS-treated perovskite surface, confirming the strengthened interfacial interaction (Figures 1E,F). We further assess the adhesion in the device stacks (ITO/SAM/perovskite/interlayer/C_60_). The low intrinsic interfacial fracture toughness at the perovskite/C_60_ interface often leads to the “weak adhesion” failure mode during delamination tests. Statistical analysis reveals that the control samples exhibit a broad distribution of fracture locations, with most fractures occurring at the perovskite/C_60_ interface. In contrast, the probability of this event is significantly reduced in the target samples, demonstrating that PTS facilitates the formation of a robust perovskite/C_60_ interface (Figure S8). To quantify the mechanical robustness of the perovskite/C_60_ interface, we measured the interfacial fracture toughness using the double cantilever beam (DCB) test. The experimental details are summarized in the Supporting Information. Consistently, the mean interfacial fracture toughness increased from 0.32 J/m^2^ for the control samples to 0.74 J/m^2^ for the target samples (Figure 1G), confirming the enhanced adhesion at the perovskite/C_60_ interface.^27,28^

### Fundamental Mechanism and Perovskite Film Properties

The enhanced interaction between perovskite and C_60_ originates from the distinctive molecular design of the PTS molecule. We calculated the electrostatic potential map of the PTS molecule, as well as the charge density of its lowest unoccupied molecular orbital (LUMO) (Figure 2A). The PTS molecule exhibits an overall electron-rich character and uniform electron delocalization across the pyrene core. As revealed by LUMO analysis, the charge localizes at the pyrene core upon electron acceptance, facilitating electron transfer to C_60_ via π-π interactions. Driven by the four sulfonate terminals, the parallel alignment of PTS on the perovskite surface is the most thermodynamically favorable configuration, thereby maximizing the interaction with C_60_ (Figure 2B). Moreover, charge density difference plots show a stronger binding affinity for C_60_ adsorption on the target surface (−0.46 eV) than on the control surface (−0.11 eV) (Figures 2C and 2D). The enhanced binding and more pronounced charge redistribution underscore the improved electron-transfer capability at the perovskite/C_60_ interface mediated by PTS. Beyond improved electrical coupling, we chose the pyrene core over smaller aromatic rings from a geometry perspective. The dimensions of pyrene are closely comparable to the diameter of the C_60_ molecule, enabling strong, continuous π-π interactions. While interactions with smaller aromatic rings can be easily disrupted, the multipoint interactions induced by pyrene provide superior stabilization and higher tolerance to minor lateral displacements, which is essential for increasing the energy barrier against C_60_ aggregation. Simulations of C_60_ lateral diffusion reveal energy barriers exceeding 0.3 eV in all directions upon interaction with the pyrene core, consistent with the suppressed aggregation in the target samples (Figure S9).

**Figure 2:**
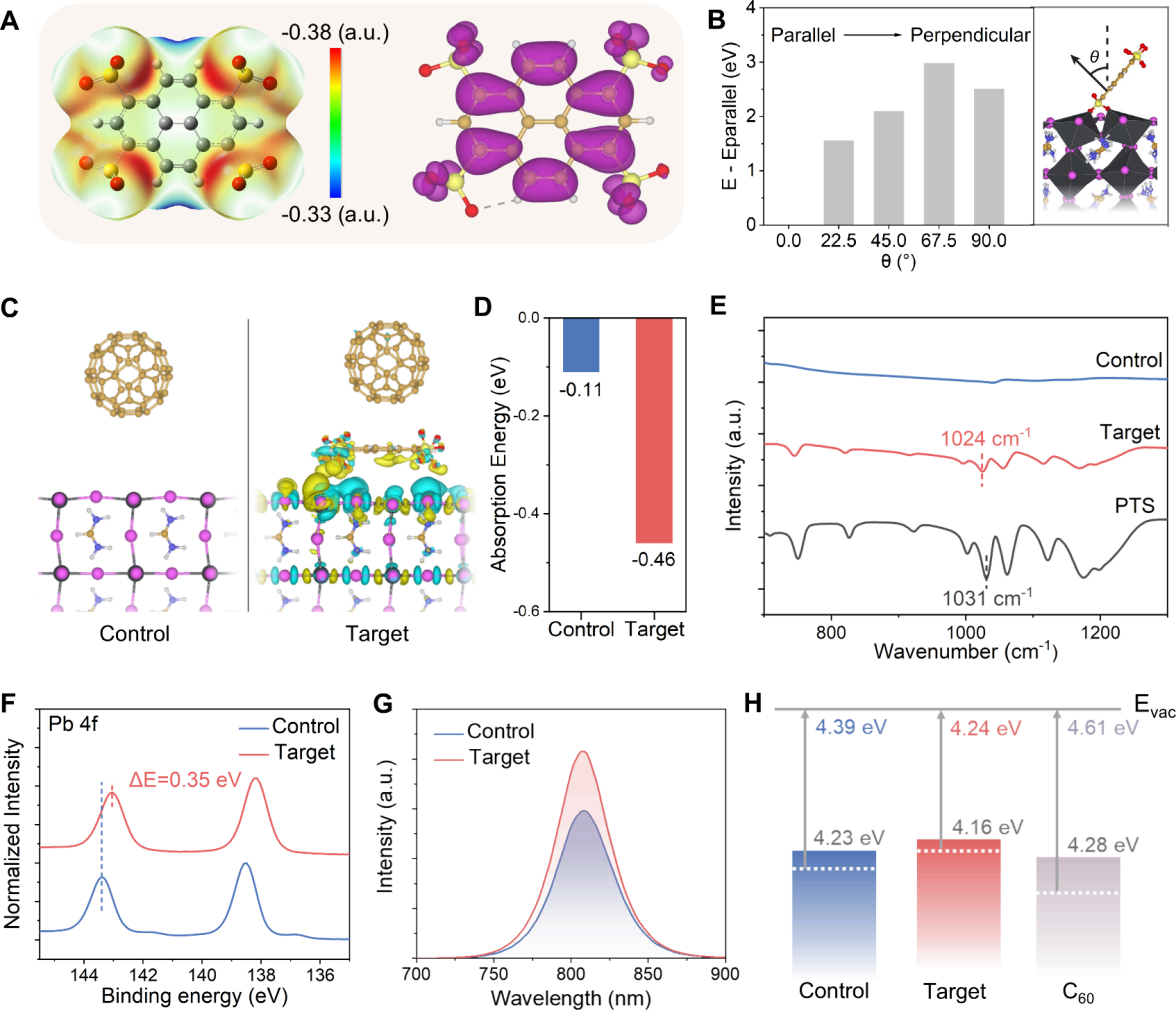
Theoretical and experimental evidence of enhanced interaction. (A) Calculated electrostatic potential (left) and LUMO charge density (right) of the PTS molecule. (B) Calculated energy difference of the PTS molecule adsorbed on the perovskite surface, ranging from parallel (set as the zero point) to the perpendicular orientations. (C) Molecular coupling of C_60_ with the control and target perovskite surfaces. (D) Calculated absorption energy of C_60_ on different surfaces. (E) Fourier transform infrared (FTIR) spectra of PTS and perovskite with and without PTS. (F) XPS spectra of Pb 4f in control and target samples. (G) PL spectra of control and target perovskite films. (H) Energy level alignment at the perovskite/C_60_ interface. Control: the film without the PTS interlayer. Target: the film with the PTS interlayer.

The interaction between PTS and perovskite was further examined through experiments. Fourier-transform infrared spectroscopy (FTIR) and X-ray photoelectron spectra (XPS) results corroborated the passivation effect of PTS (Figure 2E,F and Figure S10). The red-shifted FTIR peaks of the sulfonate group from 1,031 cm^–1^ to 1,024 cm^–1^ indicate the interaction between the sulfonate groups of PTS and perovskite, which is consistent with the 0.35 eV shift of the Pb 4*f* peak observed in XPS. Additionally, the diminished signal of Pb^0^ defects in the target film supports the passivation effect of PTS,^29,30^ while the S 2*p* peaks verify the existence of PTS in the annealed perovskite film. Steady-state photoluminescence (PL) and time-resolved PL measurements based on the quartz/perovskite structure were conducted. As illustrated in Figure 2G and Figure S11, the target perovskite films exhibit increased PL intensity and elongated carrier lifetime compared to the control films, indicating the suppressed nonradiative recombination losses. Energy level alignment is another key factor in minimizing interfacial carrier losses. UPS measurements revealed that the control perovskite film has a work function of 4.39 eV with a conduction band minimum (CBM) of 4.23 eV. For the target perovskite film, the work function shifts to 4.24 eV with a CBM of 4.16 eV (Figure 2H and Figures S12–S13). The narrower energy gap between work function and CBM indicates a stronger n-type characteristic of the target perovskite film surface, which is more favorable for efficient electron extraction at the perovskite/C_60_ interface.^31^ Consequently, PTS synergistically passivates the perovskite surface and molecularly confines C_60_, constructing a stabilized and refined perovskite/C_60_ interface with reduced energy disorder.

### Optoelectronic Stability of the Perovskite/C_60_ Interface

To validate our hypothesis, we systematically investigate the properties of the perovskite/C_60_ interface before and after aging. The samples were continuously heated at 85 °C under white LED illumination for 7 days in N_2_ atmosphere. For the perovskite layer, the PTS modification suppressed the decomposition of perovskite to PbI_2_, as evidenced by the decreased PbI_2_ signal observed in X-ray diffraction (XRD) patterns of aged samples (Figure S14). After confirming the enhanced stability of the perovskite layer, we specifically evaluate the stability of the perovskite/C_60_ stack. PL mapping measurements were conducted to analyze the evolution of nonradiative recombination under aging conditions. As shown in Figures 3A and 3B, the control sample exhibits decreased PL intensity and a broadened spatial distribution, which is attributed to severe nonradiative recombination at the perovskite/C_60_ interface. In the target sample, degradation is suppressed by PTS modification, as evidenced by more uniform emission. Kelvin probe force microscopy (KPFM) measurements reveal increased surface potential inhomogeneity in the aged control sample, while the target sample maintains uniform surface potential both before and after aging (Figure 3C,D and Figure S15). The inhomogeneous surface potential induces the formation of localized electronic traps and insufficient carrier transport at the interface. The degradation of surface potential is also observed in the perovskite/PCBM sample, which was effectively regulated by PTS (Figure S16). Additionally, the work function of the control sample is shifted by 0.62 eV, accompanied by a broadened secondary electron cutoff edge, indicating the presence of numerous defect energy levels near the Fermi level (Figure 3E). For the target sample, the work function remains nearly unchanged, with a sharp rising edge, indicating suppressed interfacial degradation.

**Figure 3:**
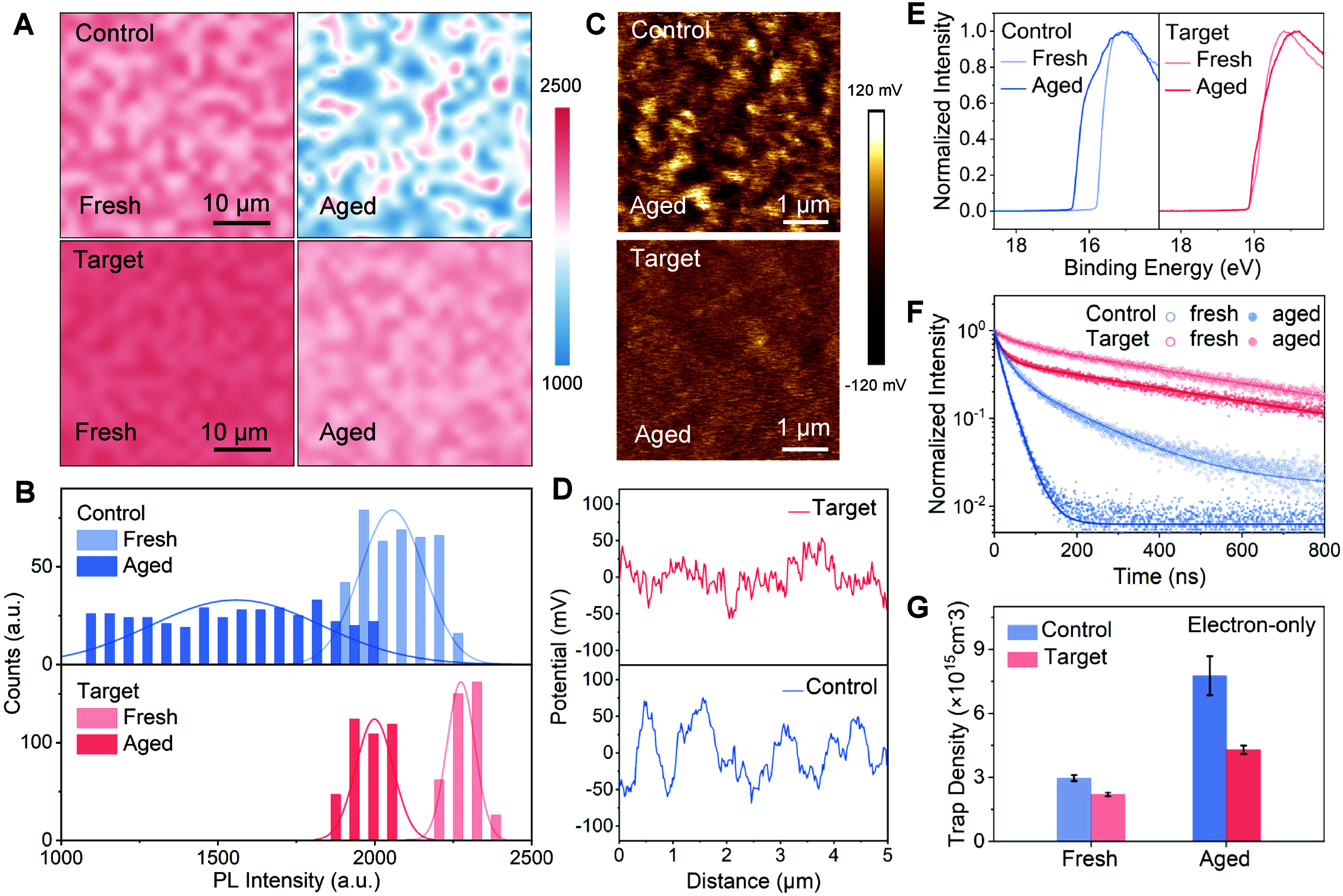
BCIS-improved optoelectronic stability of the perovskite/C_60_ interface. The samples are continuous heated at 85 °C under white LED illumination for 7 days in N_2_ atmosphere to complete the aging process. (A) PL mapping of the control and target perovskite/C_60_ films before and after aging. (B) Distribution of PL intensities in the control and target perovskite films. (C) KPFM images of the control and target perovskite/C_60_ films after aging. (D) Line profile of KPFM images. (E) Cutting-off edge of the control and target perovskite/C_60_ films. (F) TRPL results of the control and target perovskite/C_60_ films before and after aging. The solid lines show the fitting results. (G) Statistics of defect densities extracted from electron-only SCLC curves. Control: the film without the PTS interlayer. Target: the film with the PTS interlayer.

To assess the effects of the morphological and energetic inhomogeneities on carrier properties at the perovskite/C_60_ interface, we conducted time-resolved PL measurements on fresh and aged samples. As shown in Figure 3F and Table S1, the average lifetime of the control sample is significantly shortened from 132.49 to 26.22 ns, indicating increased energetic disorder and recombination at the perovskite/C_60_ interface. While the average lifetime of the target sample decreases from 564.28 to 506.30 ns, the smaller variation confirms suppressed recombination at the perovskite/C_60_ interface. Furthermore, the carrier transport properties at the interface are quantified using space-charge-limited current (SCLC) measurements based on six individual electron-only devices (Figure 3G and Figures S17–S20). The fresh target devices exhibit a lower initial trap density, with a mean of 2.2 × 10^15^ cm^–3^, compared to 2.97 × 10^15^ cm^–3^ for the fresh control devices. After aging, the mean trap density of the control devices increases significantly to 7.77 × 10^15^ cm^–3^, whereas the target devices show a smaller increase to 4.30 × 10^15^ cm^–3^. The lower trap densities in the target devices correlate with reduced interface recombination at the perovskite/C_60_ interface, thereby maintaining efficient electron transport under aging conditions. The enhanced interface stability is attributed to the superior interface integrity and the effective inhibition of C_60_ aggregation achieved by PTS modification.

### Solar Cell and Module Characteristics

To translate the observed improvements in film properties and interface stability into functional device performance, we fabricated inverted (p-i-n) PSCs with the structure ITO/NiO_*x*_/SAM/perovskite/C_60_/BCP/Ag. The current density–voltage (*J*–*V*) curves of the champion cells are shown in Figure 4A, and the device parameters are summarized in Table S2. The control device achieves a PCE of 24.66% with a *V*_oc_ of 1.144 V, a *J*_sc_ of 26.10 mA/cm^2^, and an FF of 82.59%. Notably, the target device delivers a champion PCE of 26.53% with a *V*_oc_ of 1.186 V, a *J*_sc_ of 26.12 mA/cm^2^, and an FF of 85.63%. The integrated *J*_sc_ values extracted from external quantum efficiency (EQE) measurements agree well with those from the *J*–*V* curves (Figure S21). Furthermore, the target device exhibits reduced hysteresis (Figure S22), which is attributed to enhanced electron transport at the perovskite/C_60_ interface. Under maximum-power-point (MPP) tracking, the target device exhibits a stabilized power output (SPO) of 26.07%, significantly surpassing the control device’s SPO of 23.81% (Figure 4B). Statistical analysis across 30 independent devices confirms that PTS modification primarily boosts *V*_oc_ and FF (Figures 4C and S23), consistent with suppressed interface recombination. The device performance at different concentrations of PTS is shown in Figure S24. To demonstrate the general effect of PTS, we fabricated inverted devices using PCBM as the ETL. The target device also exhibits increased *V*_oc_ and FF, consistent with the trend revealed in the C_60_-based devices. The detailed results are shown in Figure S25 and summarized in Table S3. We also employed PTS treatment in PSCs of different bandgaps and achieved performance enhancement. The champion target device based on 1.68 eV perovskite achieves a PCE of 22.67% with a *V*_oc_ of 1.252 V, while the PCE of the control device is 20.57% with a *V*_oc_ of 1.185 V (Figures S26–S27 and Table S4). For the 1.85 eV PSCs, the target device delivers a PCE of 19.04% with a *V*_oc_ of 1.363 V, compared to the control device with a PCE of 17.15% and a *V*_oc_ of 1.291 V (Figures S28–S29 and Table S5).

**Figure 4:**
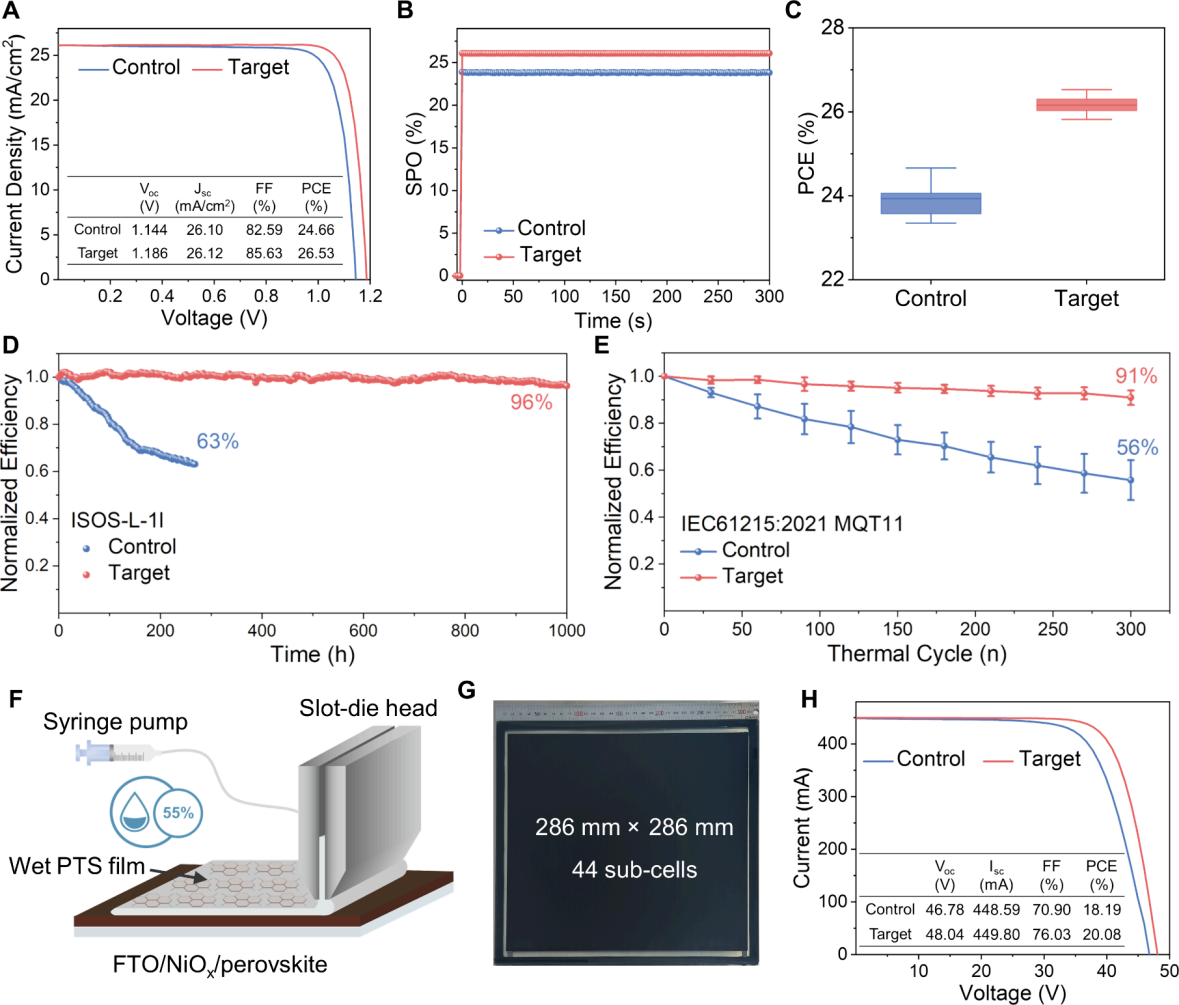
BCIS-enabled device characteristics and scalable fabrication. (A) *J*–*V* curves of devices based on 1.53 eV perovskite. (B) Steady-state power output of the control and target devices. (C) Statistical efficiency distribution of control and target devices. Thirty individual devices were used in this statistical analysis. (D) Device stability under ISOS-L-1l protocol. (E) Device stability under IEC61215:2021 MQT11 protocol with temperature ranges from −40 to 85 °C. (F) Schematic illustration of the slot-die coating of PTS on perovskite film. (G) Image of the perovskite module fabricated under RH 55%. (H) *J–V* curve of the control and target perovskite module. Control: the film without the PTS interlayer. Target: the film with the PTS interlayer.

We further assessed the device stability following standardized protocols. The target device retains 96% of its initial efficiency after 1,000 h of continuous MPP operation (ISOS-L-1l), whereas the control device rapidly degrades, losing 37% of its initial efficiency within the first 300 h (Figure 4D). To assess the impact of interfacial toughening on the thermo-mechanical stability of devices, we conduct thermal cycling tests in accordance with the IEC 61215:2021 MQT11 standard (from −40 to 85 °C, with a cycle duration of 5.5 h). After 300 cycles, the control device retains only 56% of its initial efficiency, whereas the target device maintains 91% of its original performance (Figure 4E). The enhanced device stabilities highlight the effectiveness of interface toughening by PTS.

Finally, we evaluate the scalability of the BCIS effect by employing PTS on perovskite solar modules (PSMs) via slot-die coating, as schematically illustrated in Figure 4F. PSMs are fabricated in ambient air (relative humidity, RH, 55%) using industrially-compatible manufacturing facilities. Figure 4G shows a photograph of a typical perovskite solar module with an aperture area of 818 cm^2^ and 44 subcells. We confirm uniform passivation across different regions of large perovskite films based on PL measurements. As shown in Figure S30, the PL intensity of the control film exhibits large fluctuations among 9 positions, while the target film shows enhanced average PL intensity with a narrower distribution. A similar conclusion can be drawn from the EL images of perovskite modules, in which the target module exhibits more uniform EL emission (Figure S31). Despite the high relative humidity in the fabrication environment, the target module achieves a PCE of 20.08%, a *V*_oc_ of 48.04 V, an *I*_sc_ of 449.80 mA, and an FF of 76.03%. In comparison, the control module delivers a PCE of 18.19% with a *V*_oc_ of 46.78 V, an *I*_sc_ of 448.59 mA, and an FF of 70.90% (Figure 4H). It is worth noting that we are amongst the first to report PSMs with PCE over 20% and an aperture area over 800 cm^2^, fabricated at over 50% humidity. In contrast, in the literature, high-PCE PSMs of comparable size are mostly fabricated at much lower RH^32–35^ (Table S6). The enhanced photovoltaic parameters further demonstrate the effectiveness of PTS and verify the pivotal role of our strategy in the cell-to-module, scale-up fabrication of PSCs.

## Conclusions and Outlook

With the PTS interlayer, we reconfigured the perovskite/C_60_ interface to mitigate the critical impact of chemo-mechanical degradation at the heterointerfaces in inverted PSCs. Multidentate anchoring of sulfonate groups and geometry-guided π-π interactions of the pyrene core enhance the interaction and adhesion between perovskite and C_60_ while suppressing the aggregation of C_60_ molecules, thereby achieving the molecular BCIS effect. The improved properties and device performance attest to the positive role of the BCIS, while the processing ease of the PTS interlayer makes BCIS inherently scalable. This work establishes BCIS as an effective approach for maintaining molecular-level interface integrity in stable PSCs. We envision that fundamental research along this line will complement existing compositional and encapsulation strategies while offering a new perspective on stabilizing PSCs and commercial solar modules.

## Supplementary Material



## Notes

Materials and methods; experimental details; DFT calculation details; Supplementary Note; and characterization data including SEM, AFM, UPS, UV–vis, XPS, TRPL, XRD, SCLC, EQE, J-V, EL, and device performance parameters (PDF)
