# Supplementary material for: Bidirectional Chemo-Mechanical Interface Stabilization in Perovskite Solar Cells
Source: J Am Chem Soc. 2026 Jul 16;148(29):31146–53. doi: 10.1021/jacs.6c06932 (PMC13426265; doi:10.1021/jacs.6c06932)
Supplement: Supplementary file 1 [file ja6c06932_si_001.pdf]

Supporting Information

Bidirectional chemo-mechanical interface stabilization in perovskite solar cells

Qian Cheng,[1, ‡] Xiaofen Li,[1, ‡], Mingwei Hao,[1], Kuan Wang,[1] Jiahong Tang,[1] Pengfei Guo,[1] Lifei He,[1] Wenjian Yu,[1] Changyu Yang,[1] Du Chen,[2,3] Peijun Guo,[2,3] Yuanyuan Zhou[1,4,5,*]

Materials and Methods; experimental details; DFT calculation details; Supplementary Note; and characterization data including SEM, AFM, UPS, UV-vis, XPS, TRPL, XRD, SCLC, EQE, J-V, EL and device performance parameters.

Materials and Methods

Raw chemicals

Dimethylformamide (DMF, 99.8%), dimethyl sulfoxide (DMSO, 99.9%), chlorobenzene (CB, 99.8%), ethanol (99.8%), isopropanol (IPA, ≥99.8%), and PbI_2 were acquired from Sigma-Aldrich. $NH_2CH{=}NH_2I$ (FAI, >99.99%), $NH_2CH{=}NH_2Br$ (FABr, >99.99%), CH_3NH_3Cl (MACl, >99.99%), and CH_3NH_3Br (MABr, >99.99%) were purchased from Greatcell Solar. $PbBr_2$ (99.998%), CsI (99.998%), propylammonium chloride (PACl), C_{60} (99.5%), PCBM (99.5%) and bathocuproine (BCP) (99%) were acquired from Xi'an Yuri Solar Co., Ltd. NiO_x nanopowder was purchased from Advanced Election Technology Co., Ltd. Me-4PACz and MeO-2PACz were acquired from TCI. 1,3,6,8-Pyrenetetrasulfonic acid tetrasodium salt (PTS)was purchased from Aladdin. All raw chemicals were used as received without further purification.

Preparation of solutions

The NiO_x solution was prepared by dispersing 10 mg of NiO_x powder in a 1 mL mixture of H_2O-IPA (v/v, 3:1). The solution was ultrasonicated for 15 min and then filtered through a 0.22 μm filter before use. The SnO_2 solution was diluted five times with deionized water before use. Both the Me-4PACz and MeO-2PACz solutions were dissolved in ethanol at 0.3 mg/mL, and the solutions were mixed at a 2:1 volume ratio before use. For the 1.53 eV perovskite solution, 1.6 M $FA_{0.95}Cs_{0.05}PbI_3$ with 10% MACl and 10% excess PbI_2 was dissolved in 1 mL mixed DMF-DMSO (4:1, v/v) and stirred for 2 h. For the 1.68 eV perovskite solution, 1.45 M $FA_{0.8}MA_{0.15}Cs_{0.05}PbI_{2.34}Br_{0.66}$ with 7% excess PbI_2 was dissolved in 1 mL mixed DMF-DMSO (4:1, v/v) and stirred for 2 h. For the 1.85 eV, 1.2 M $FA_{0.7}MA_{0.1}Cs_{0.2}PbI_{1.5}Br_{1.5}$ with 3% excess of PbI_2 was dissolved in 1 m L of a mixed DMF-DMSO (4:1, v/v) and stirred for 2 h. 0.3 mg of PTS was dissolved in 1 mL of mixed IPA-DMF (v/v, 150:1). Prior to use, the solution was sonicated for 20 min and then filtered through a 0.22 μm PTFE syringe filter. 20 mg PCBM was dissolved in 1 mL CB and stirred overnight to form the PCBM solution, which was then filtered through a 0.22 μm PTFE syringe filter before use. 0.5 mg BCP was dissolved in 1 mL IPA and stirred overnight to form the BCP solution.

Material characterizations

The surface morphology was measured using a multi-mode 8 AFM platform (Brucker) using a 0.01–0.025 Ω cm antimony (n-)doped Si tip (RTESPA-300) in a non-contact tapping mode. The KPFM was performed with a 0.01–0.025 Ω cm antimony (n-)doped Si tip (SCM-PIT-V2) in a PeakForce-KPFM mode. The SEM images were collected using a JSM-6700F (JEOL) microscope. XRD was performed using X'pert Pro (PANalytical) with Cu Kα radiation (λ = 1.5406 Å) in the θ-2θ scan mode. The absorption spectra of the thin films were measured using a Lambda 1050+ spectrophotometer (PerkinElmer). The PL mapping measurements were performed using an InVia Qontor (Renishaw Ltd.) with a 532 nm laser. For the TRPL measurements, the sample was excited with a 375 nm picosecond laser (LDH-D-C-375,

PicoQuant), and the counts were collected using a single-photon avalanche photodiode. TRPL spectra were fitted with a double exponential decay function: $f(t) = A_1 \exp(-t/\tau_1) + A_2 \exp(-t/\tau_2)$, where A_1 and A_2 are the decay amplitudes, and τ_1 and τ_2 are the decay time constants. FTIR spectra were collected using a Vertex 70 Hyperion 1000 (Bruker). X-ray photoelectron spectroscopy (XPS) and ultraviolet photoelectron spectroscopy (UPS) measurements were performed using Axis Supra+ (Kratos Analytical Limited). A dual-beam focused ion beam (FIB) nanofabrication tool (FEI Helios5 UC) was employed to prepare cross-sectional TEM samples. TEM samples were investigated by using a Thermo Scientific Spectra 300 (S)TEM microscope at 300 kV.

Mechanical reliability tests

The delamination tests were performed on samples with the structure ITO/perovskite/C_{60}/UV glue/glass. After the deposition of C_{60}, the UV glue was applied and cured under UV light for 30 s to form a reliable contact. Finally, an upward force was applied to the cover glass, causing the samples to delaminate. To delaminate perovskite single crystals, the evaporated C60 layer was removed with adhesive tape.

For the measurement of interfacial fracture toughness, the "sandwich" double cantilever beam (DCB) specimens were prepared with the structure glass/ITO/SAM/perovskite/C_{60}/ALD-SnO_2/Cu/epoxy/glass, with ITO-coated glass of dimensions 12.5 mm x 37.5 mm x 1.1 mm. The ITO-coated glass and blank glass slide used for the sandwich structure have identical thicknesses (1.1 mm). The epoxy used was a 5-minute Loctite Epoxy, which was left to cure at room temperature for 24 h before mechanical testing. Edges of the specimens were cleaned with a razor blade to reduce the effect of epoxy "bridging" between the two glass beams and skewing test results. A tape was used to cover both ends of the sample before depositing C_{60}. After curing, a blade was inserted along the edge of the tape to create a "pre-crack" to help initiate crack propagation. DCB mechanical testing [1] [2] was conducted using a delaminator adhesion test system (DTS, Menlo Park, CA). Tension and compressive forces were applied to the sample to perform the loading-unloading cycle. The displacement rate was set to 1 μm/s while simultaneously recording the load (P) and displacement (Δ). The crack length (a) was estimated using the following equation:

$$a = \left(\frac{d\Delta}{dP} * \frac{BEh^3}{8}\right)^{\frac{1}{3}} - 0.64h$$

where B is the width of the glass substrate, E is the Young's modulus of the glass substrate, and h is the thickness of the sample. In our condition, *B* is 12.5 mm, *E* is 70 GPa, and *h* is 1.1 mm.

The interfacial fracture toughness is then given with the following equation:

$$G_C = \frac{12{P_C}^2a^2}{B^2Eh^3}\left(1 + 0.64\frac{h}{a}\right)^2$$

where P_C is the load at the onset of nonlinearity in the P–Δ curve. The loading-unloading cycles were repeated 5 times, and the GC values were calculated as the average of these repetitions.

DFT calculation

Density functional theory (DFT) calculations were performed using the Vienna Ab initio Simulation Package (VASP). The projector-augmented wave (PAW) method was employed to accurately describe electron–ion interactions. Generalized gradient approximation of Perdew-Burke-Ernzerhof (PBE) was utilized, and a kinetic energy cutoff of 500 eV was applied. Gaussian smearing with a width of 0.05 eV was adopted in the calculations. Both lattice parameters and atomic positions were fully relaxed until the residual forces on each atom were less than 0.02 eV $Å^{-1}$. For the calculations of the properties of a single additive molecule, Γ-centered 2 × 2 × 2 *k*-meshes were used. For the calculations of the interaction characteristics between the perovskite supercell, additive molecules, and C60, Γ-centered 1 × 1 × 1 k-meshes were used, with a vacuum layer thickness greater than 15 Å in all interface models.

Solar cell fabrication and testing

ITO substrates were sequentially cleaned by sonication with deionized water and IPA for 15 min each. All substrates were treated with UV-ozone for 15 min to improve the wettability. 60 μL NiO_x solution was spin-coated onto the ITO substrate at 4000 rpm for 30 s, followed by annealing at 150°C for 10 min in ambient conditions. Then the substrates were transferred into the N_2 glove box. The mixed SAM solution was spin-coated at 3000 rpm for 30 s, and annealed at 100°C for 10 min. For the 1.53 eV perovskite, 120 μL of the precursor solution was spread onto the substrate, followed by a two-stage spin-coating process (1000 rpm for 10 s and then 4800 rpm for 40 s). 260 μL chlorobenzene was dropped onto the substrate 8 s before the end of the spin-coating. The wet perovskite films were immediately annealed at 100 °C for 40 min. For the 1.68 eV perovskite, 100 μL of the precursor solution was spread onto the substrate and spun at 1000 rpm for 10 s, then at 5000 rpm for 30 s. 260 μL of chlorobenzene was added to the substrate 10 s before the end of the spin-coating. The wet perovskite films were immediately annealed at 100 °C for 20 min. For the 1.85 eV perovskite, 100 μL of the precursor solution was spread onto the substrate, then spun at 2000 rpm for 10 s, followed by 5500 rpm for 40 s. 260 μL chlorobenzene was dropped onto the substrate 20 s before the end of the spin-coating. The wet perovskite films were immediately annealed at 100 °C for 20 min. After the substrates were cooled, the PTS layer was dynamically spin-coated onto the perovskite layer at 5000 rpm for 30 s, followed by annealing at 100°C for 10 min. The temperature of the glove box was controlled at 20~23 °C during the fabrication. Then the substrates were transferred into a thermal evaporation chamber to deposit the C_{60} layer (20 nm), the BCP layer (7 nm), and the Ag electrode (100 nm) under vacuum. For the PCBM-based device, the PCBM solution was spin-coated onto the as-fabricated perovskite substrate at 2000 rpm for 30s, followed by annealing at 70°C for 5 min. The BCP layer was cast by spin-coating the BCP solution at 6000 rpm for 30s, followed by annealing at 70°C for 2 min. Then the

Ag electrode was thermally evaporated onto the substrates to complete the device. During *J-V* testing, the active device area is defined by a 0.069 cm^2 mask. For the electron-only device, the SnO_2 solution was spin-coated onto the ITO substrate at 3000 rpm for 30 s and annealed at 150°C for 30 min in ambient air. The following fabrication processes were identical to those for the regular device.

The *J–V* characterization for small-area PSCs was measured by a source meter (2612, Keithley) under AM 1.5 G one-sun illumination (100 mW cm^{-2}) generated by a class AAA solar simulator (Sirius-SS, Zolix) in a nitrogen-filled glovebox. A high-precision metal mask was used to define the active area for each measurement. The reference cell used to calibrate the solar simulator before *J–V* measurements was an Oriel reference solar cell accredited by NIST to the ISO-17025 standard. The 1.53 eV PSCs were tested in the voltage range of 1.2 V to -0.1 V at the reverse direction (from V_{oc} to J_{sc}) with a step size of 0.02 V and a dwell time of 10 ms. As for the 1.68 eV and 1.85 eV PSCs, the voltage range was changed to 1.3 V to -0.1 V and 1.4 V to -0.1 V. External quantum efficiency (EQE) spectra were recorded at a chopping frequency of 165 Hz in AC mode using a solar cell quantum efficiency measurement system (QE-R3011, Enlitech) to ensure the reliability of the *J-V*-determined short-circuit densities of our best-performing devices. For the SCLC analysis, the scan range was -1 V to 4 V with a step size of 0.02 V under dark conditions. Steady-state power outputs were measured using the 2612 source meter (Keithley) at the voltages determined from the MPPs of the reverse-scan *J–V* curves. The operational stability was tested under continuous LED illumination and MPP tracking. For the thermal cycling stability test, the devices were placed in a −40 °C to 85 °C cycled environment controlled by a programmable temperature chamber, with each cycle lasting 5.5 h.

Solar module fabrication and testing

The 320 mm × 320 mm FTO substrates were first marked with four points at 15 W, 45 ns pulse width, and 50 kHz repetition rate (P0). Then the P1 line was laser-scribed on the FTO substrates using the same parameters as P0, resulting in a scribe width of 19.23 μm. The patterned FTO substrates were cleaned three times by sonication in deionized water, then dried with nitrogen gas. Before use, the FTO was treated with UV ozone for 10 min. A 14-nm NiO_x layer was deposited by physical vapor deposition (PVD). Before coating the perovskite layer, the substrates were transferred to a UV-ozone chamber for 5 min. The perovskite solution was slot-die-coated onto the FTO/NiO_x substrate with a 39 μm gap, a pump dispense rate of 23.5 μL/s, and a coating speed of 20 mm/s. After coating, the wet film was immediately transferred to the vacuum chamber dryer (VCD) to remove the solvent at 0.5 Pa. Then, the substrate was annealed at 110°C for 25 min. For the PTS layer, the solution was slot-die-coated onto the perovskite with a 40 μm gap, a pump dispense rate of 30 μL/s, and a coating speed of 30 mm/s using an N_2 knife, followed by annealing at 100°C for 5 min. Afterward, 15 nm C_{60} and 20 nm SnO_2 were deposited on the perovskite through thermal evaporation and atomic layer deposition (ALD). The P2 line was laser-scribed using a 532 nm laser with 26 W of power, an 80 ns pulse width, and a 134 kHz repetition rate, yielding a scribe width of 35.23 μm. Then, 80 nm of Cu was also deposited as the top contact using PVD. The P3 line was first

scribed using a 532 nm laser with a power of 21 W, a pulse width of 30 ns, and a repetition rate of 18 kHz to clean the Cu layer, and then a 532 nm laser with a power of 27 W, a pulse width of 30 ns, and a repetition rate of 130 kHz to clean the bottom layer. The width of the P3 line is 32.28 μm. For PSMs, the *J-V* characterization was measured using a source meter (2460, Keithley) under AM 1.5 G one-sun illumination (100 mW cm^{-2}) generated by a class AAA solar simulator (L-400, Qingdao Solar Scientific Instrument High-tech Co., LTD). The perovskite modules were tested over a voltage range of 49 V to -1 V in reverse direction (from V_{oc} to J_{sc}), acquiring 50 data points.

Note S1. The potential molecular design beyond PTS.

From our perspective, the ideal interfacial molecules should be bifunctional, combining an anchoring part and a conjugated part to enhance interaction between the perovskite and C_{60}. The orientation of molecules on the perovskite surface is also important. Parallel stacking on the perovskite substrate could maximize π-π interactions with C_{60} and minimize interfacial electrical resistance, thereby ensuring efficient charge transport at the interface. Based on these requirements, future molecular engineering can proceed in several promising directions. The properties of the interfacial materials can be tuned by modifying the conjugated core and the anchoring groups. For example, altering the size of the conjugated system could optimize carrier mobility, while employing different anchoring groups could change the binding strength of the molecule and the electron density in the conjugated core. To go beyond non-covalent interactions, synthesizing molecules that can react with perovskite and modified C_{60} may form a chemically bonded interface, benefiting ultra-stable, efficient perovskite devices.

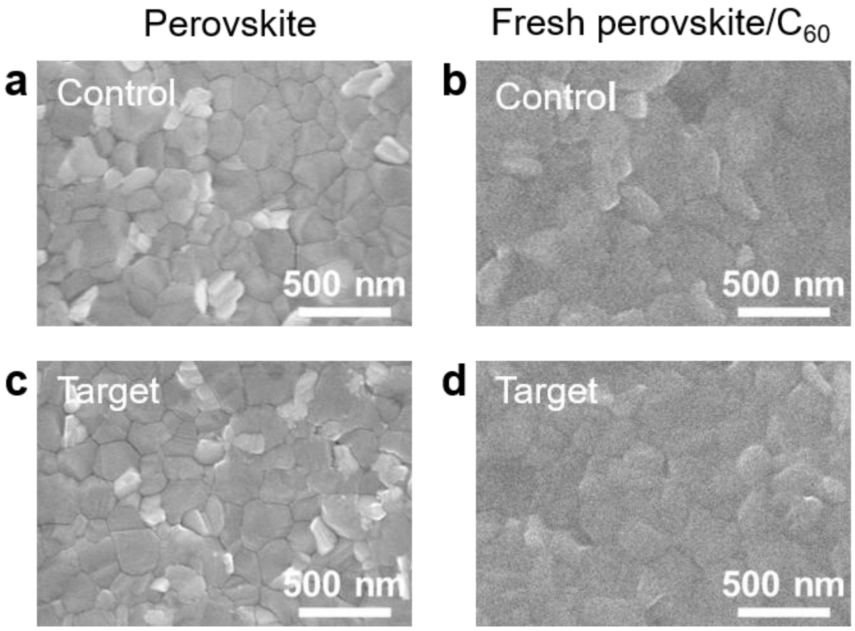

Figure S1. SEM images of (a, c) perovskite, (b, d) fresh perovskite/C_{60}. As shown in the SEM images, both the fresh control and target perovskite films are fully covered by evaporated C_{60}.

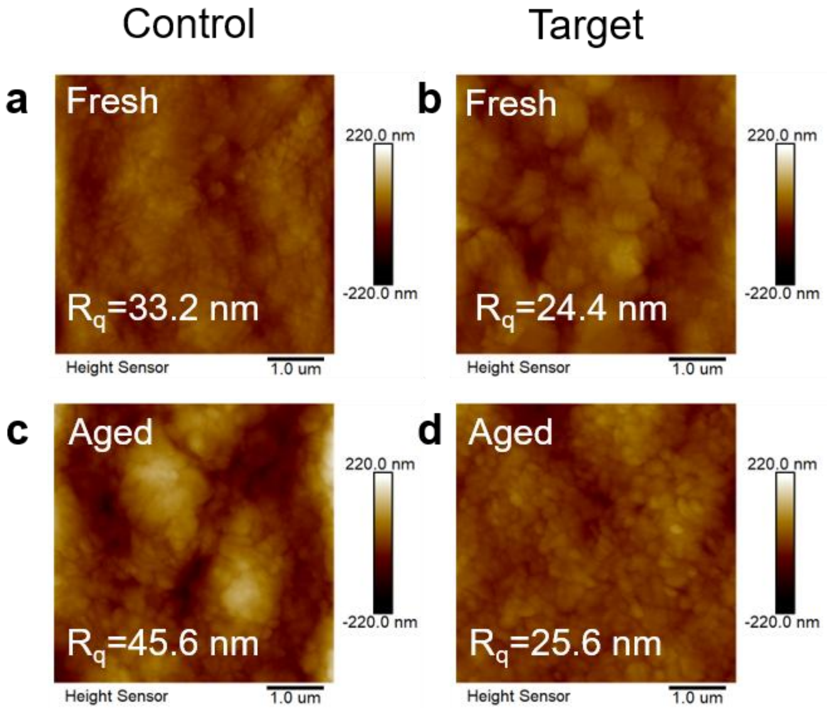

Figure S2. AFM images of control and target perovskite/C_{60} films (a,b) before and (c,d) after photo-thermal aging. Both fresh control and target films are uniform, with decreased roughness in the target film. After photo-thermal aging, the control film exhibits several micrometer-scale protrusions, whereas the target film remains flat. The well-maintained morphology arises from enhanced interactions among perovskite, PTS, and C_{60}.

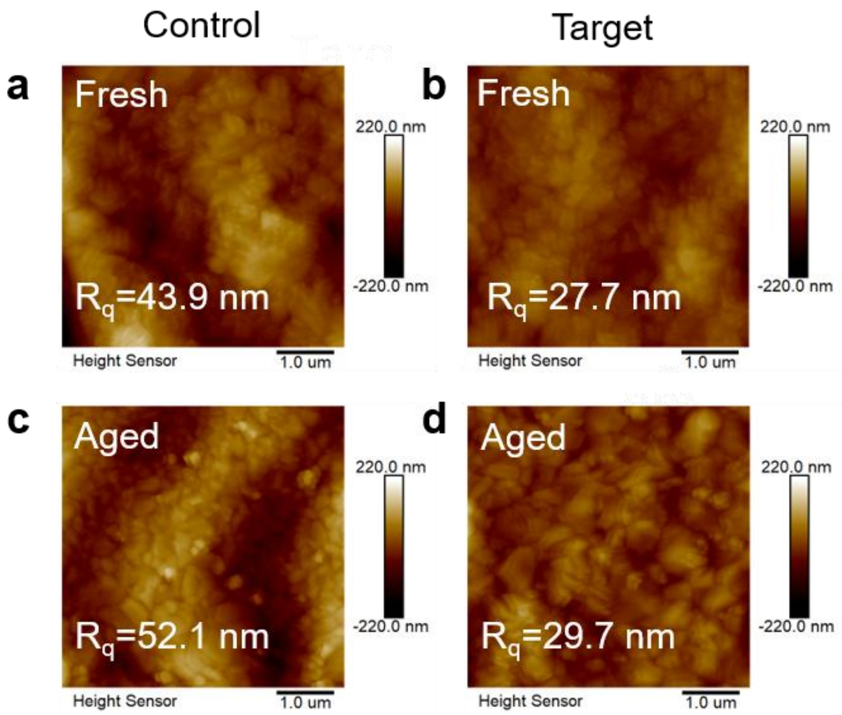

Figure S3. AFM images of control and target perovskite films (a,b) before and (c,d) after photo-thermal aging. Compared with the perovskite/C60 film, the bare perovskite film surface is rougher. After photo-thermal aging, the control film exhibits ridge-like protrusions, demonstrating that the phenomena observed in the aged control perovskite/C_{60} film arises from aggregated C_{60}. For the target film, the surface remains smooth, and the degradation is inconspicuous, supporting the protection of the interface by the PTS interlayer.

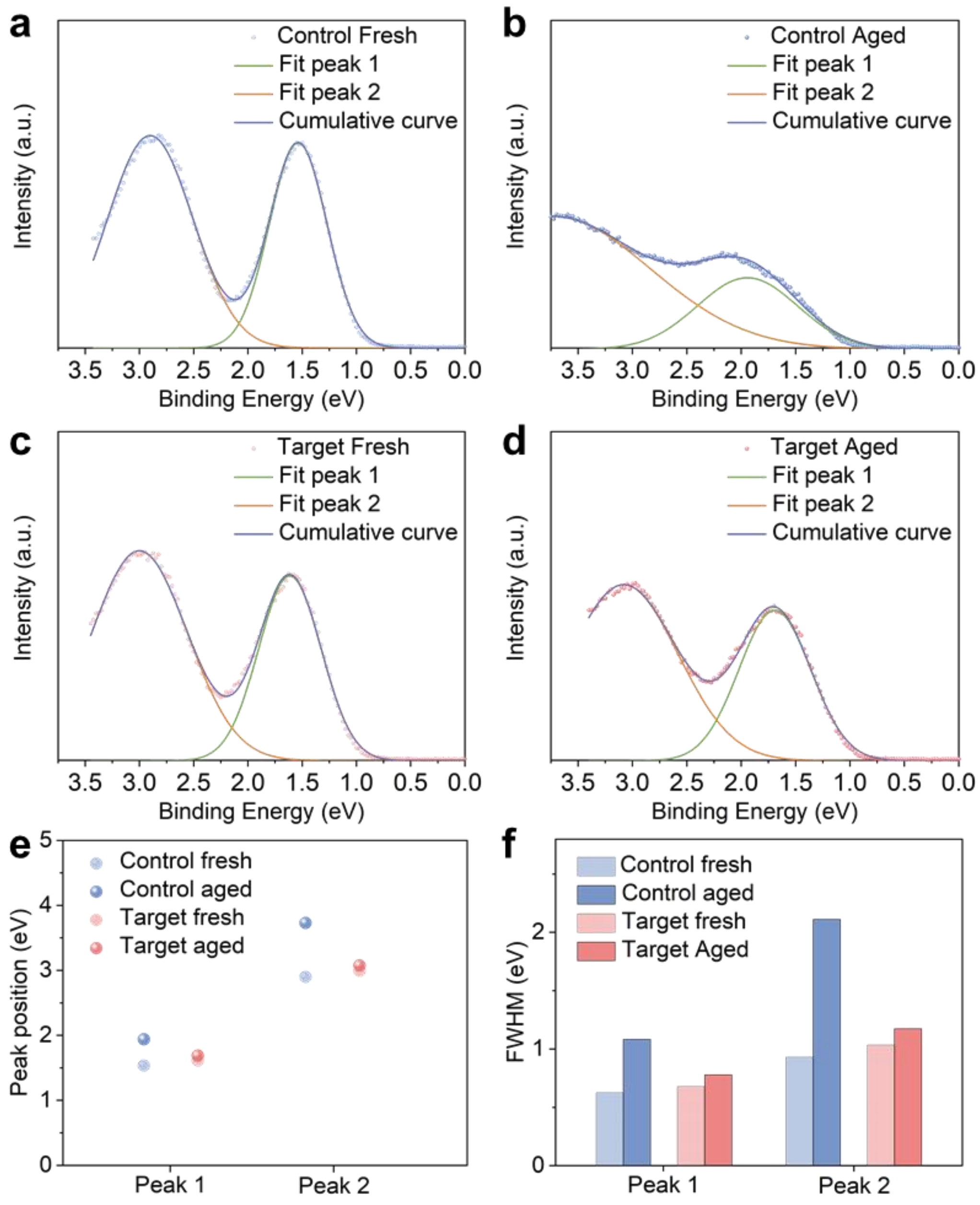

Figure S4. a-d, Peak analysis of UPS spectra, including a, fresh and b, aged control perovskite/C_{60} sample, c, fresh and d, aged target perovskite/C_{60} sample. The evolution of e, peak position, and f, FWHM variation extracted from the UPS peaks.

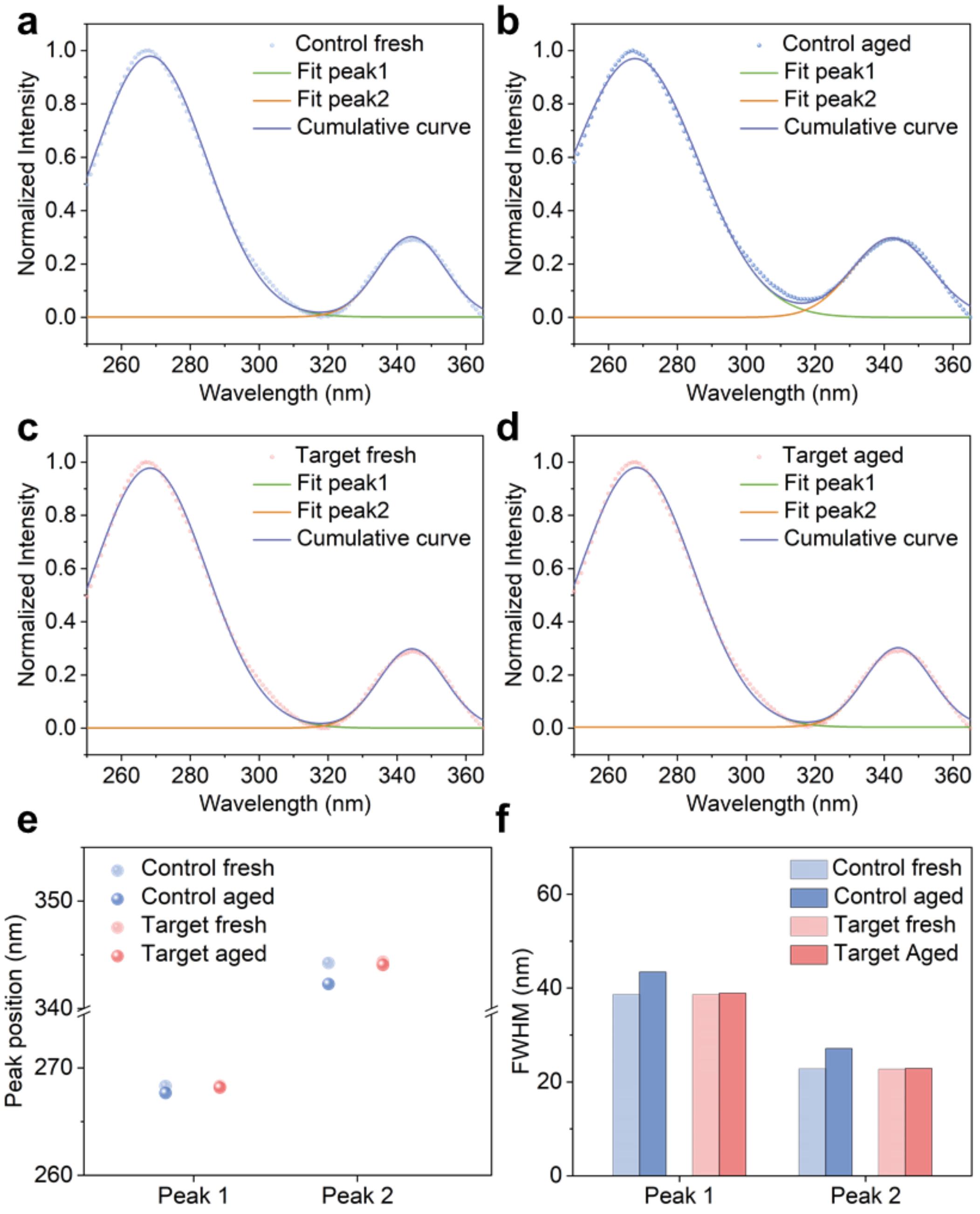

Figure S5. a-d, Peak analysis of UV-vis absorption spectra, including a, fresh and b, aged control C_{60} sample, c, fresh and d, aged target C_{60} sample. The evolution of e, peak position, and f, FWHM variation extracted from the absorption peaks.

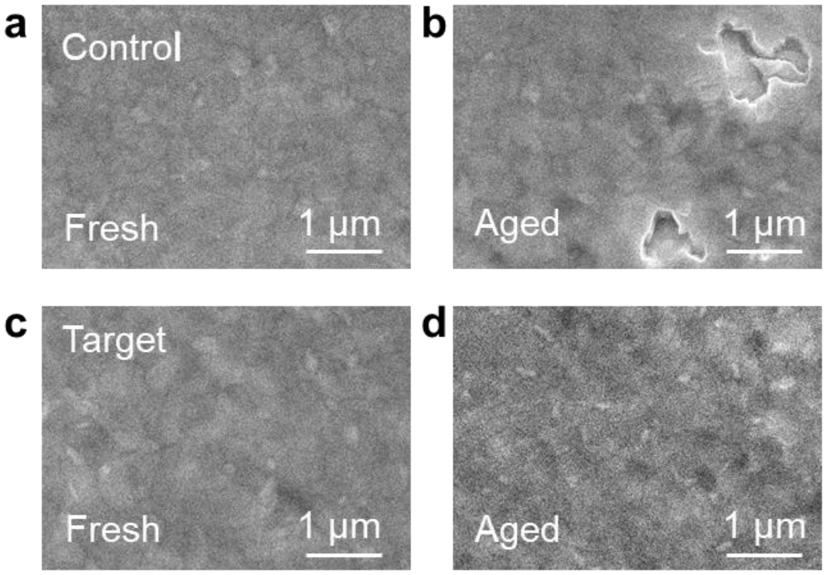

Figure S6. SEM images of (a, b) control and (c, d) perovskite/PCBM samples. As shown in the SEM images, both the fresh control and target perovskite films are fully covered by PCBM. After aging, there are several cracks in the control sample while the target sample shows slight degradation.

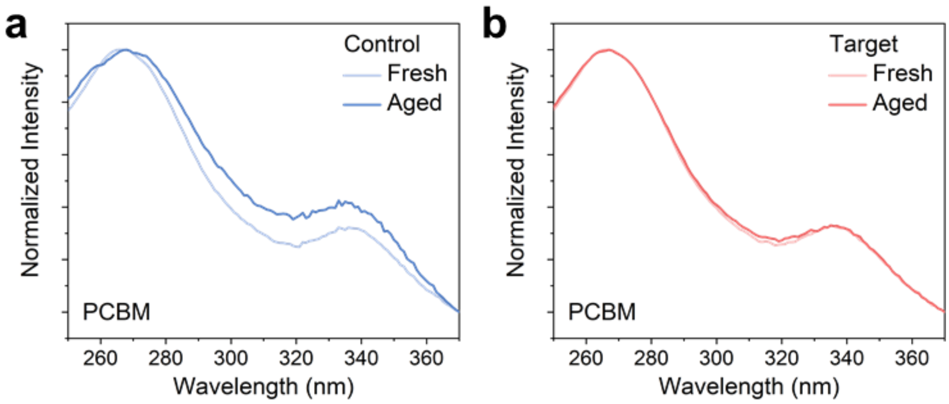

Figure S7. UV-vis absorption spectra of a) control and b) target PCBM films before and after photo-thermal aging. The results are similar to those of the C60 films, which also indicate suppressed molecular aggregation during aging.

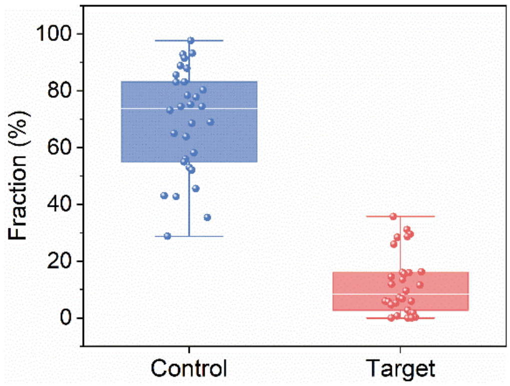

Figure S8. Statistics of the perovskite/C_{60} interface delamination test results based on 30 samples for both control and target cases.

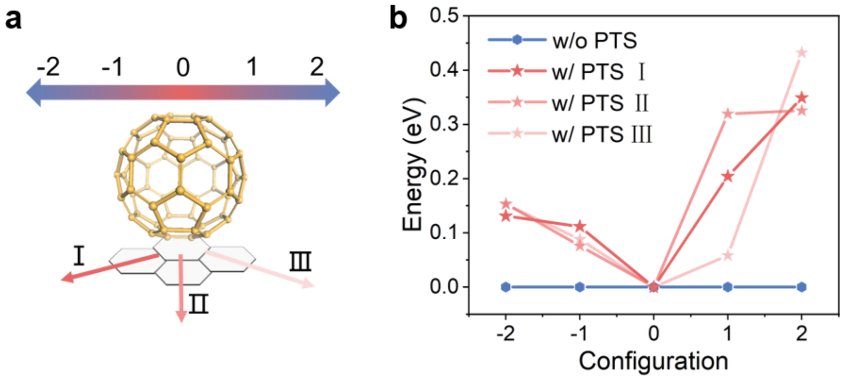

Figure S9. Energy barriers for the motion of C_{60} along different directions (I, II, III) on PTS. Although the change in total energy varies slightly across other directions, energy barriers exist in all cases, indicating the formation of a three-dimensional energy barrier in the actual situation.

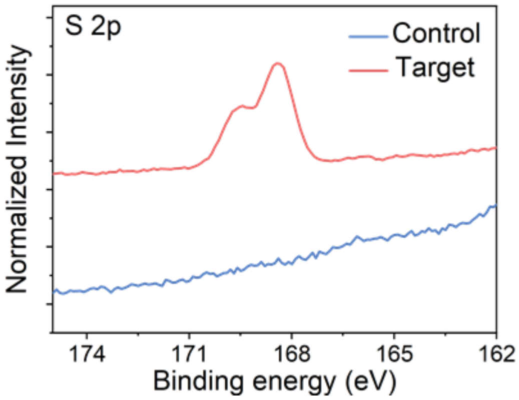

Figure S10. S 2p XPS spectra of the control and target perovskite films. The observed S 2p peak proves the existence of PTS at the surface of the perovskite film.

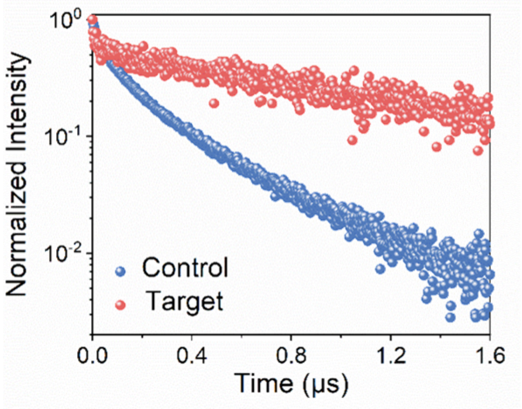

Figure S11. TRPL results of the control and target perovskite films. The elongated lifetime in the target sample proves the suppressed recombination by the PTS treatment.

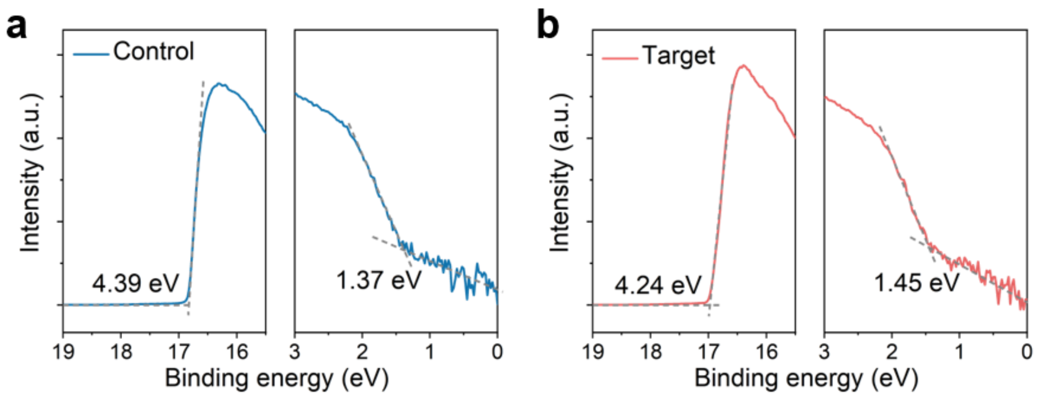

Figure S12. UPS spectra of the control and target perovskite films. The results reveal that the control perovskite film has a work function of 4.39 eV with a conduction band minimum (CBM) of 4.23 eV. Induced by PTS, the work function shifts to 4.24 eV with a CBM of 4.16 eV for the target perovskite film. The up-shifted work function and n-type surface facilitate the electron transfer at the perovskite/C_{60} interface, leading to suppressed interfacial recombination.

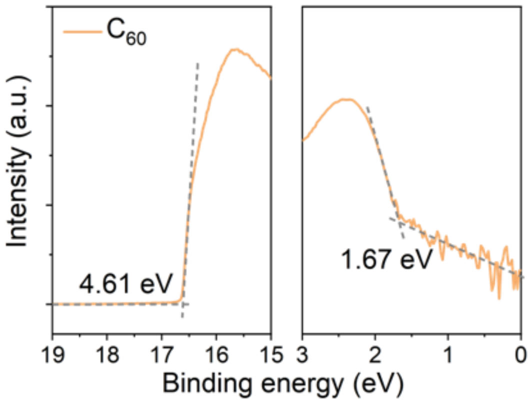

Figure S13. UPS spectra of the fresh ITO/C_{60} film. The work function of C_{60} is 4.61 eV with a CBM of 4.28 eV. Appropriate energy alignment between perovskite and C_{60} is essential for efficient electron transfer at the interface.

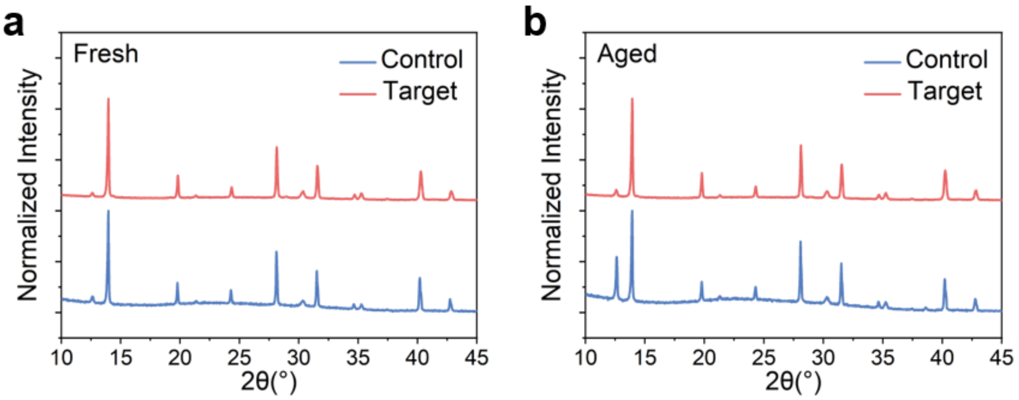

Figure S14. XRD patterns of the control and target perovskite films a, before and b, after photo-thermal aging. For the control perovskite film, the increased PbI_2 peak is attributed to perovskite degradation, which is suppressed in the target film by the PTS interlayer.

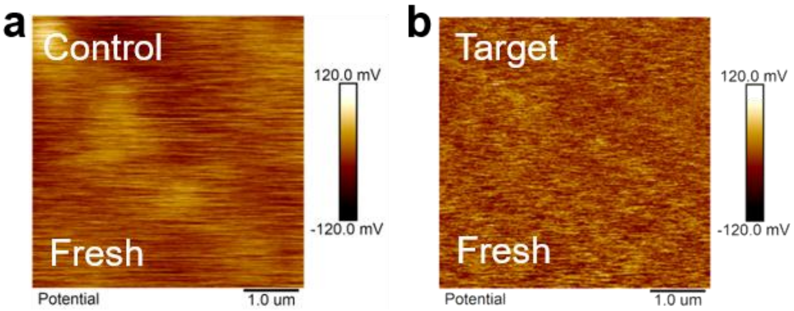

Figure S15. KPFM images of fresh control and target perovskite/C_{60} films. The surface potential of fresh target perovskite films is more uniform than that of the control film, demonstrating the homogenized electrical properties induced by the PTS interlayer.

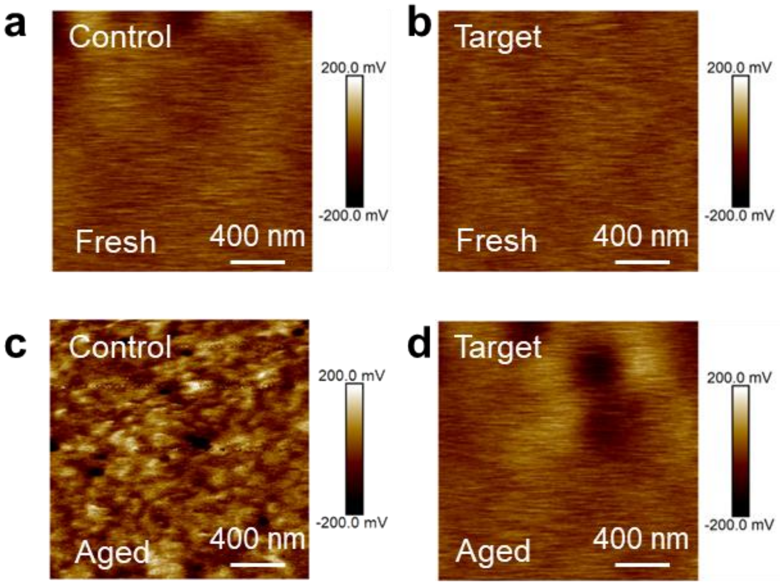

Figure S16. KPFM images of the control and target perovskite/PCBM films before and after aging. The surface potential of fresh target perovskite films is slightly more uniform than that of the control film. After aging, the control film exhibits a random surface potential due to PCBM aggregation, whereas only part of the target film is degraded. The experiments based on PCBM further confirm the stabilization induced by PTS.

Table S1. Fitting parameters of control and target perovskite/C_{60} films before and after aging.

		A_1	τ_1 (ns)	A_2	τ_2 (ns)	τ_{avg} (ns)
Control	fresh	0.61	26.65	0.33	164.24	132.49
	aged	0.32	9.39	0.66	28.87	26.22
Target	fresh	0.25	55.86	0.73	581.02	564.28
	aged	0.44	21.57	0.43	526.62	506.30

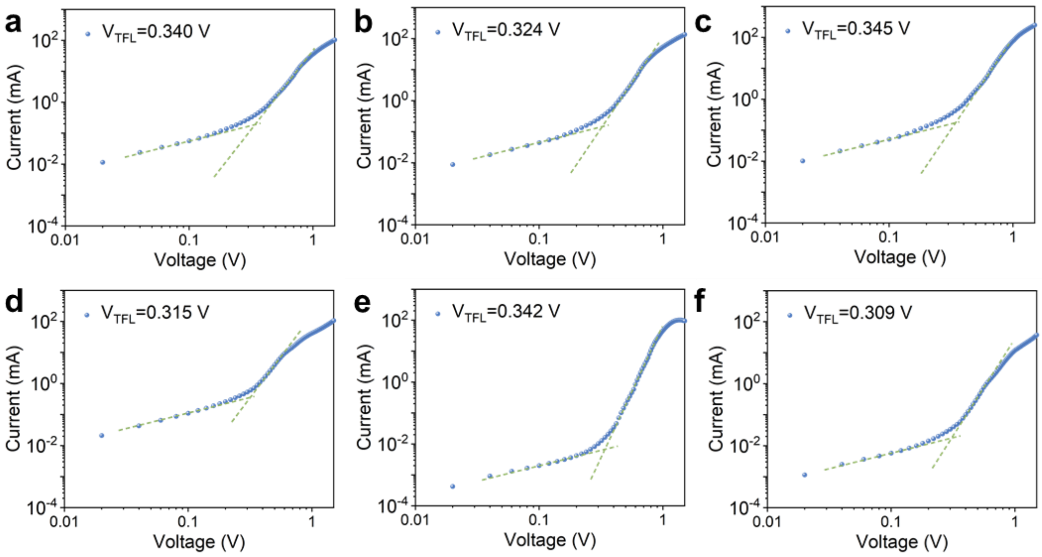

Figure S17. SCLC curves of 6 individual fresh control electron-only devices.

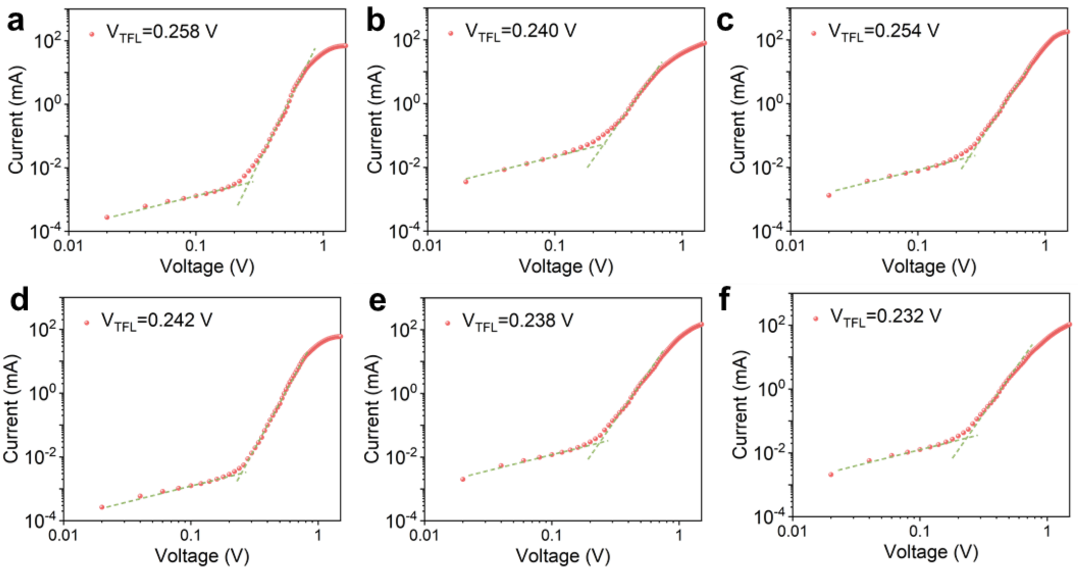

Figure S18. SCLC curves of 6 individual fresh target electron-only devices. In the fresh cases, the reduced V_{TFL} on the target device indicates suppressed recombination at the perovskite/C_{60} interface by PTS.

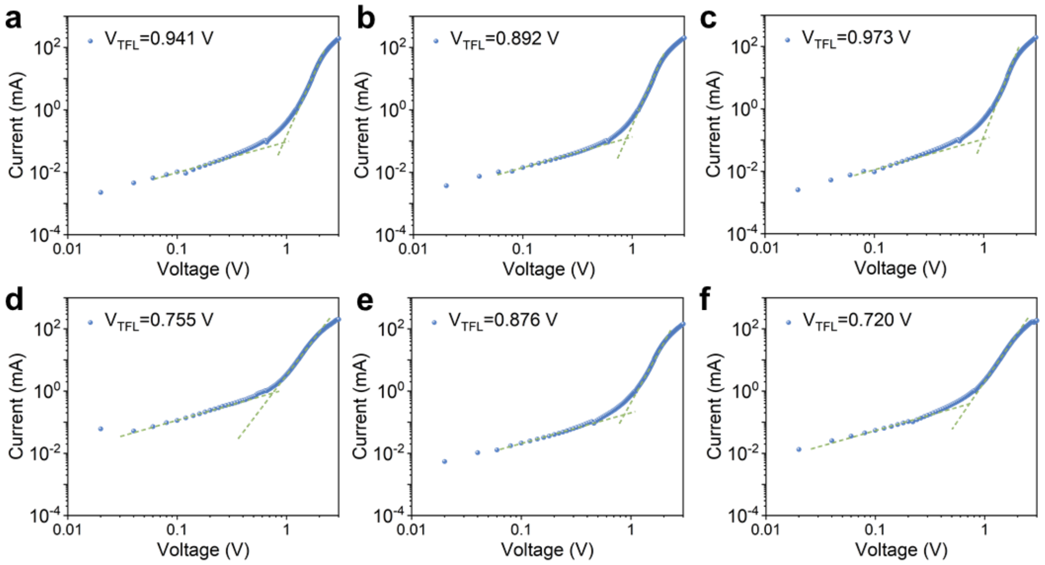

Figure S19. SCLC curves of the aged control electron-only devices.

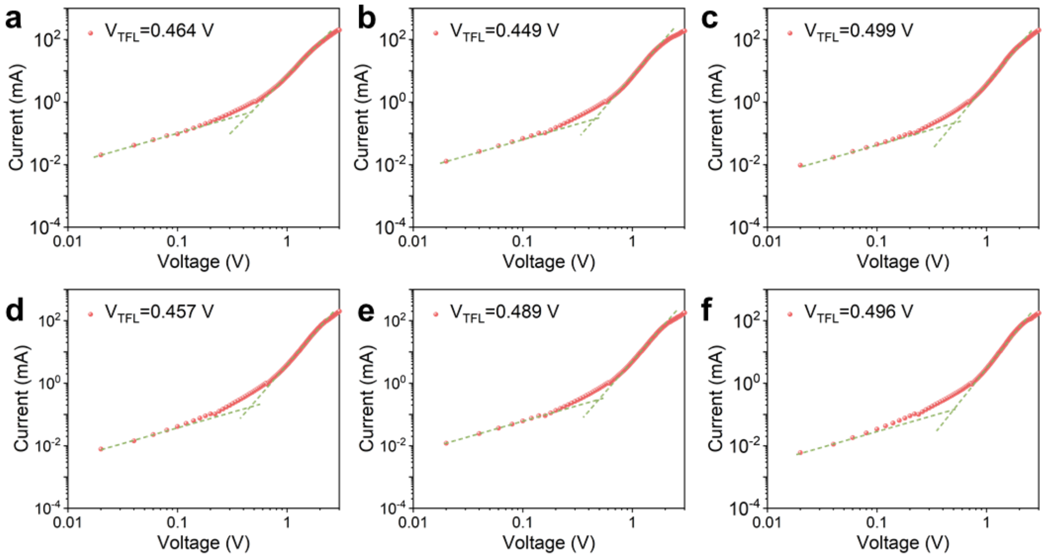

Figure S20. SCLC curves of aged target electron-only devices. After aging, both V_{TFL} in the control and target devices increase, indicating an affected electron transfer at the degraded interface. The lower increase in trap density in the target devices demonstrates that PTS stabilizes the perovskite/C_{60} interface, thereby enabling efficient electron transport under aging conditions.

Table S2. Device parameters of control and target perovskite solar cells.

	Scan direction	V_{oc} (V)	J_{sc} (mA/cm^2)	FF (%)	PCE (%)
**Control**	Reverse	1.144	26.10	82.59	24.66
	Forward	1.135	26.11	80.18	23.76
**Target**	Reverse	1.186	26.12	85.63	26.53
	Forward	1.183	26.13	84.97	26.26

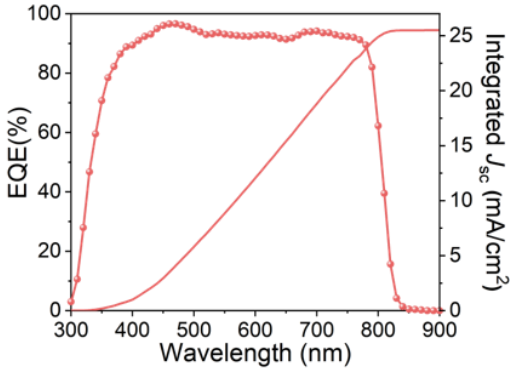

Figure S21. EQE spectra of the champion device. The integrated J_{sc} is 25.50 mA/cm^2, which agrees with the J_{sc} in the J-V curve.

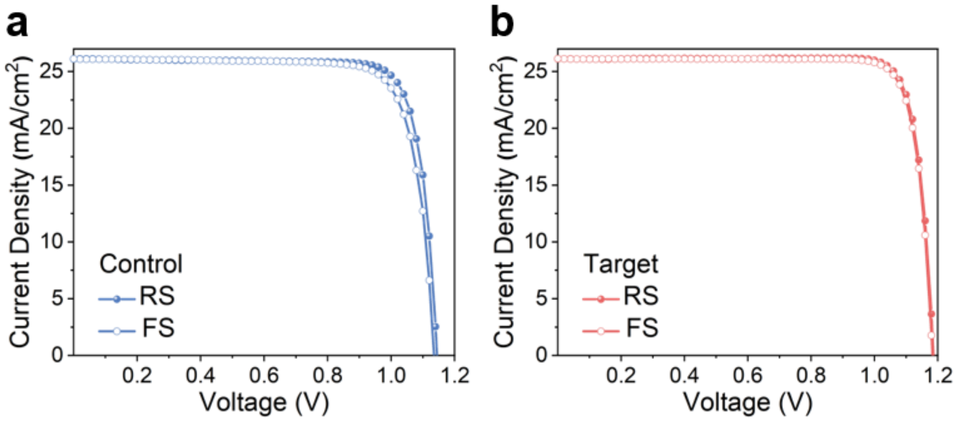

Figure S22. *J-V* curves of the a, control and b, target devices under reverse scan (RS) and forward scan (FS). HI= ($PCE_{reverse}$-$PCE_{forward}$)/$PCE_{reverse}$. The hysteresis index (HI) is calculated as the difference between the reverse and forward scans: 3.6% for the control device and 1% for the target device. The reduced hysteresis stems from more efficient electron transfer and reduced carrier accumulation at the perovskite/C_{60} interface.

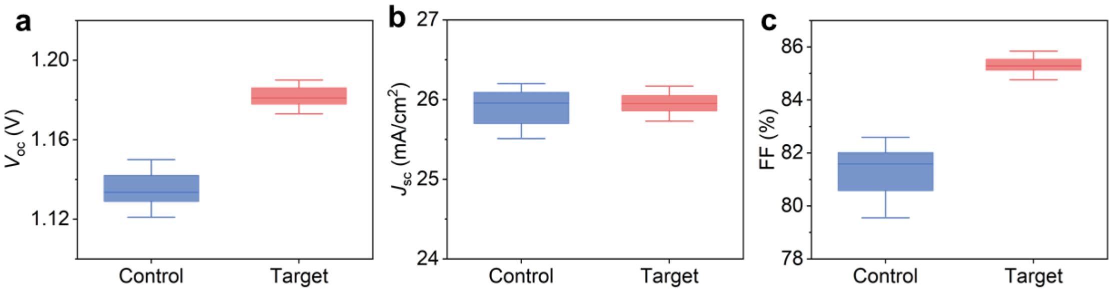

Figure S23. Statistics of a, V_{oc}, b, J_{sc}, c, FF of control and target devices. 30 individual devices of each condition are used in this analysis. The results show that the main improved device parameters are *Voc* and FF, arising from suppressed recombination and facilitated electron transfer via the PTS interlayer.

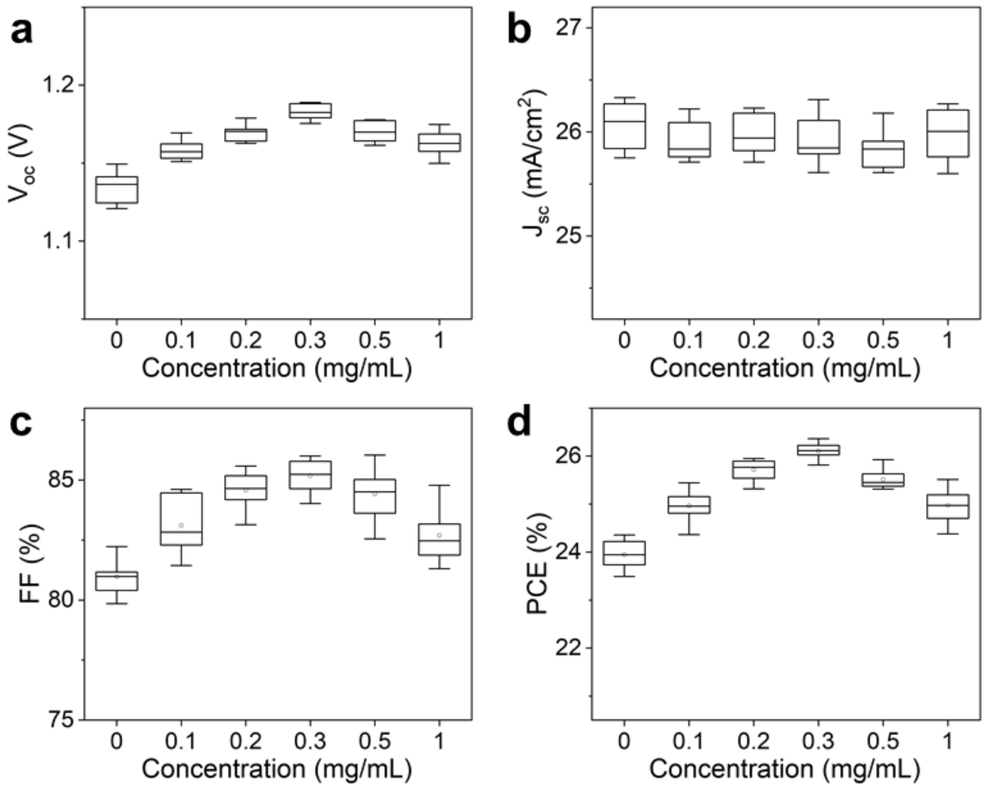

Figure S24. Statistics of a, V_{oc}, b, J_{sc}, c, FF, d, PCE of devices employing PTS modification with different concentrations. 10 devices per concentration are used in this analysis.

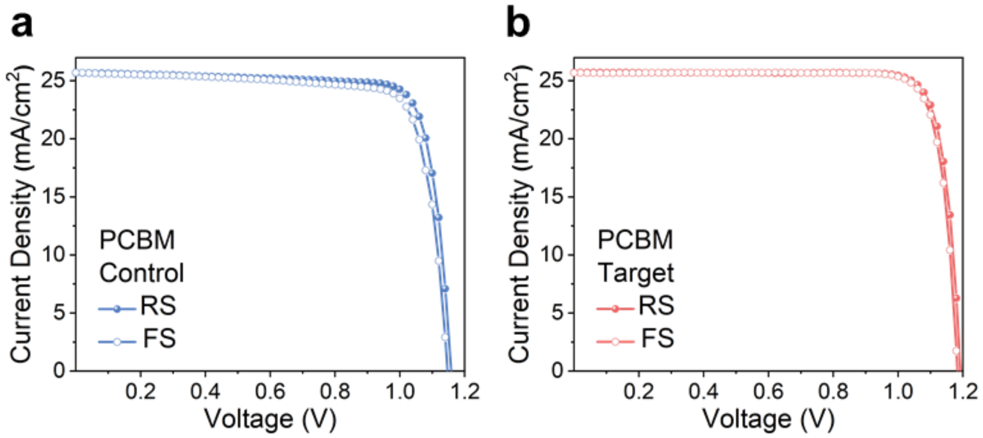

Figure S25. *J-V* curves of the a, control, and b, target devices based on PCBM as ETL under reverse scan (RS) and forward scan (FS). The improved performance proves the general effect of PTS.

Table S3. Device parameters of control and target devices based on PCBM ETL.

	Scan direction	V_{oc} (V)	J_{sc} (mA/cm^2)	FF (%)	PCE (%)
**Control**	Reverse	1.158	25.69	81.56	24.26
	Forward	1.147	25.70	79.65	23.48
**Target**	Reverse	1.192	25.70	85.25	26.11
	Forward	1.183	25.70	84.97	25.83

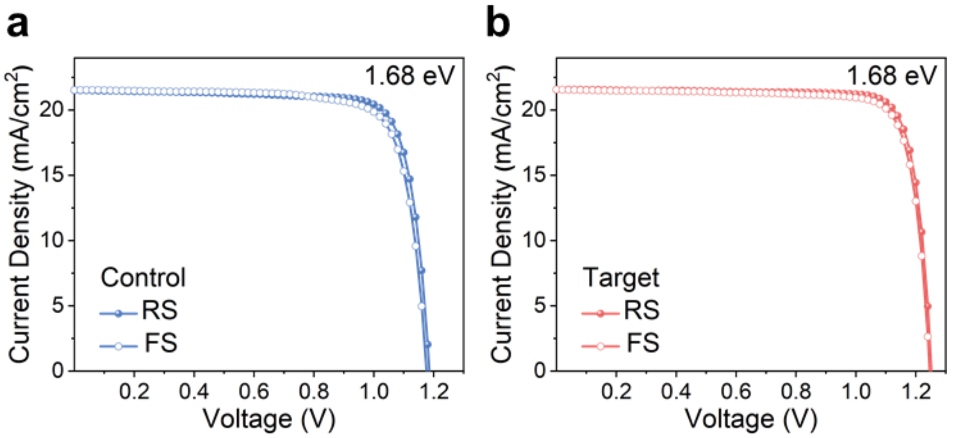

Figure S26. *J-V* curves of the a, control, and b, target devices based on 1.68 eV perovskite under reverse scan (RS) and forward scan (FS). The reduced hysteresis comes from the enhanced carrier properties at the perovskite/C_{60} interface.

Table S4. Device parameters of control and target devices based on 1.68 eV perovskite.

	Scan direction	V_{oc} (V)	J_{sc} (mA/cm^2)	FF (%)	PCE (%)
**Control**	Reverse	1.185	21.51	80.70	20.57
	Forward	1.176	21.52	78.55	19.88
**Target**	Reverse	1.252	21.55	84.02	22.67
	Forward	1.246	21.56	82.34	22.12

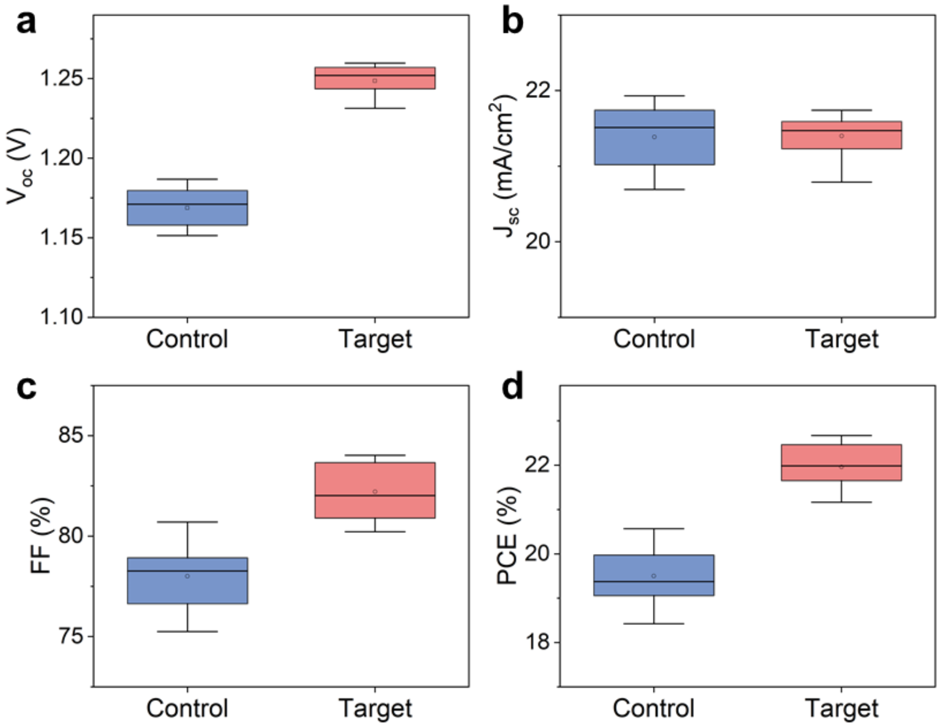

Figure S27. Statistics of a, V_{oc}, b, J_{sc}, c, FF, d, PCE of control and target devices based on 1.68 eV perovskite. 15 devices per condition are used in this analysis.

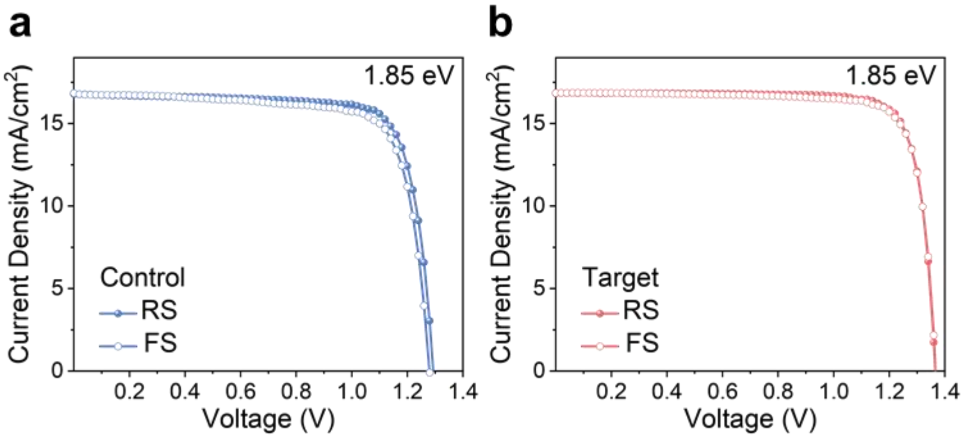

Figure S28. *J*-*V* curves of the a, control and b, target devices based on 1.85 eV perovskite under reverse scan (RS) and forward scan (FS). The reduced hysteresis comes from the enhanced carrier properties at the perovskite/C_{60} interface.

Table S5. Device parameters of control and target devices based on 1.85 eV perovskite.

	**Scan direction**	**V_{oc} (V)**	**J_{sc} (mA/cm^2)**	**FF (%)**	**PCE (%)**
**Control**	Reverse	1.291	16.78	79.17	17.15
	Forward	1.277	16.79	76.91	16.49
**Target**	Reverse	1.363	16.82	83.05	19.04
	Forward	1.363	16.84	82.14	18.85

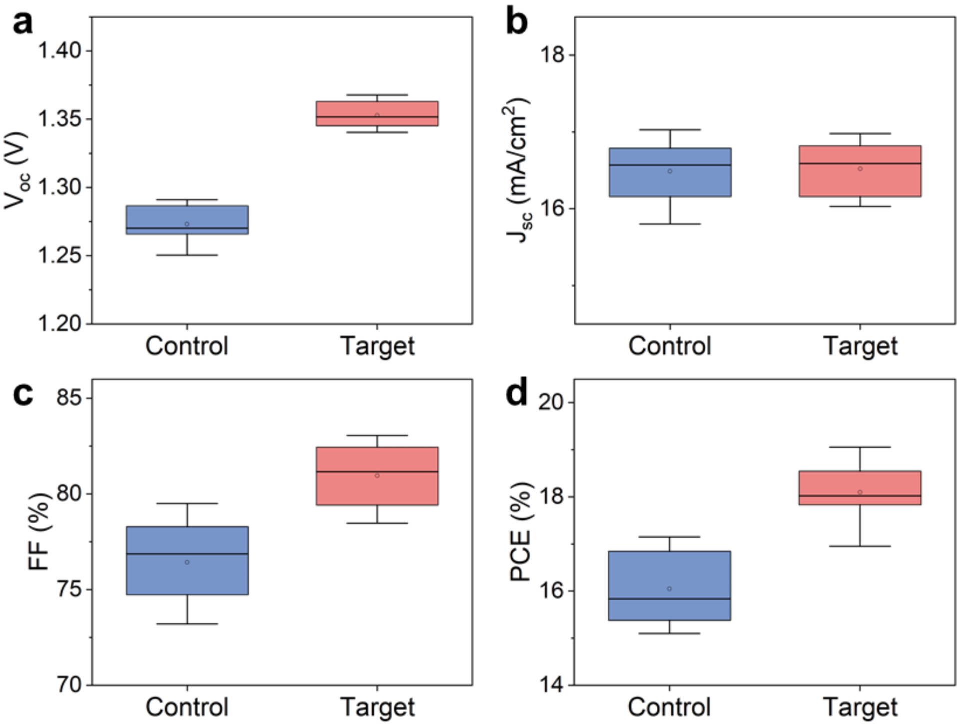

Figure S29. Statistics of a, V_{oc}, b, J_{sc}, c, FF, d, PCE of control and target devices based on 1.85 eV perovskite. 15 devices per condition are used in this analysis.

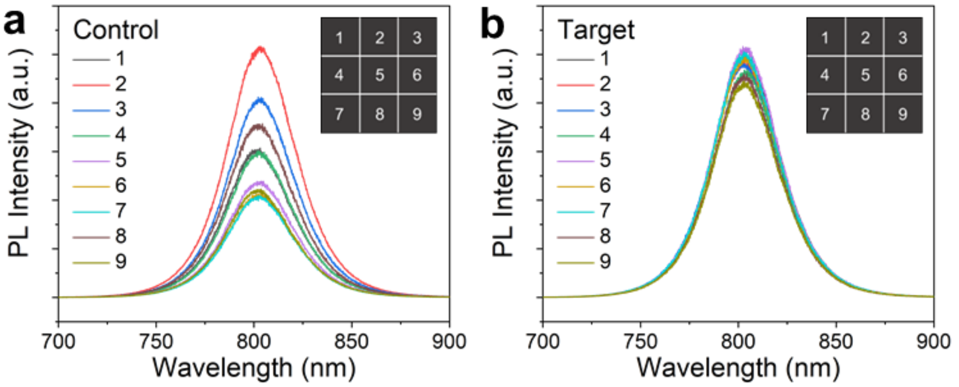

Figure S30. PL spectra of a, control and b, target large perovskite films at 9 selected positions.

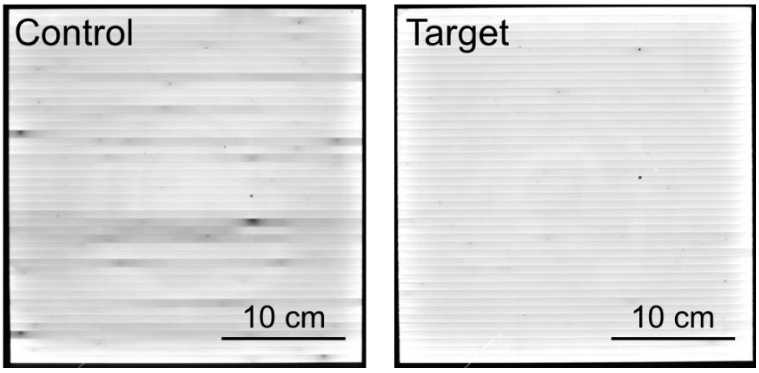

Figure S31. EL images of control and target perovskite modules.

Table S6. Summary of perovskite solar modules with an area over 700 cm^2.

Strategy	Area (cm^2)	Efficiency (%)	Ref
Suppression of PCBM dimer	764	21.3	[3]
Vapor-assisted surface reconstruction	785	19.6	[4]
Solvent engineering	1017.5	21.4	[5]
	7200	18.0	
Matrix-confined HTL	20000	20.05	[6]
3D laminar flow–assisted crystallization	7906	15	[7]
In situ coordinated HTL	749.3	20.21	[8]
Impurity-healing interface	715.1	22.46	[9]
low-dimensional perovskite passivation	802	17.59	[10]
Bidirectional chemo-mechanical interface stabilization	818	20.08	This work

Reference

1. Dai, Z.; Yadavalli, S. K.; Chen, M.; Abbaspourtamijani, A.; Qi, Y.; Padture, N. P., Interfacial toughening with self-assembled monolayers enhances perovskite solar cell reliability. *Science* **2021,** *372* (6542), 618-622.
2. Dong, B.; Wei, M.; Li, Y.; Yang, Y.; Ma, W.; Zhang, Y.; Ran, Y.; Cui, M.; Su, Z.; Fan, Q., Self-assembled bilayer for perovskite solar cells with improved tolerance against thermal stresses. *Nat. Energy* **2025,** *10* (3), 342-353.
3. Liang, Z.; Xu, H.; Huang, Z.; Lei, X.; Ye, J.; Zhang, Y.; Zhu, P.; Liu, B.; Chen, W.; Wang, X., Suppression of PCBM dimer formation in inverted perovskite solar cells. *Nat. Mater.* **2025,** *25*, 267-274.
4. Sun, X.; Shi, W.; Liu, T.; Cheng, J.; Wang, X.; Xu, P.; Zhang, W.; Zhao, X.; Guo, W., Vapor-assisted surface reconstruction enables outdoor-stable perovskite solar modules. *Science* **2025,** *388* (6750), 957-963.

5. Wang, Y.; Liu, Y.; Luo, X.; Xiao, K.; Marrugat-Arnal, V.; Xu, D.; Lou, J.; Zuo, W.; Tavakoli, N.; Li, T., Improved solvent systems for commercially viable perovskite photovoltaic modules. *Science* **2025,** *390* (6777), 1021-1028.
6. Liang, Y.; Chen, G.; Wang, Y.; Zou, Y.; Feng, M.; Wang, Y.; Li, B.; Cho, Y.; Chang, Y.; Liu, T., A matrix-confined molecular layer for perovskite photovoltaic modules. *Nature* **2025,** *648* (8092), 91-96.
7. Yan, B.; Dai, W.; Wang, Z.; Zhong, Z.; Zhang, L.; Yu, M.; Zhou, Q.; Ma, Q.; Yan, K.; Zhang, L., 3D laminar flow–assisted crystallization of perovskites for square meter–sized solar modules. *Science* **2025,** *388* (6749), eadt5001.
8. Sun, Y.; Xu, R.; Dai, J.; Tang, H.; Wang, J.; Cai, W.; Li, P.; Xu, J.; Yuan, F.; Jiao, B., In situ coordinated HTL strategy for high-performance and scalable perovskite solar cells. *Nat. Commun.* **2025,** *16* (1), 9110.
9. Wang, H.; Su, S.; Chen, Y.; Ren, M.; Wang, S.; Wang, Y.; Zhu, C.; Miao, Y.; Ouyang, C.; Zhao, Y., Impurity-healing interface engineering for efficient perovskite submodules. *Nature* **2024,** *634* (8036), 1091-1095.
10. Li, J.; Jin, C.; Jiang, R.; Su, J.; Tian, T.; Yin, C.; Meng, J.; Kou, Z.; Bai, S.; Müller-Buschbaum, P., Homogeneous coverage of the low-dimensional perovskite passivation layer for formamidinium–caesium perovskite solar modules. *Nat. Energy* **2024,** *9* (12), 1540-1550.
